# Refining the NEED (Needs Examination, Evaluation and Dissemination) Methods for Use in Psychotic Disorders in Belgium: Lessons From the Design Phase

**DOI:** 10.1111/hex.70724

**Published:** 2026-08-31

**Authors:** Zilke Claessens, Io Wens, Emma Olieslaeger, Ruud Van Winkel, Pierre Oswald, Margaux Sageot, Vicky Steyfkens, Rani Claerman, Robby De Pauw, Laurence Kohn, Caroline Obyn, Julie Paradis, Claudia Schönborn, Irina Cleemput, Charline Maertens de Noordhout, Isabelle Huys

**Affiliations:** ^1^ Department of Pharmaceutical and Pharmacological Sciences Regulatory Science Research Group, KU Leuven Leuven Belgium; ^2^ Research Foundation Flanders Brussel Belgium; ^3^ Department of Neurosciences Center for Clinical Psychiatry, KU Leuven Leuven Belgium; ^4^ University Psychiatric Center (UPC) KU Leuven Leuven Belgium; ^5^ Department of Psychiatry Brussels University Hospital Brussels Belgium; ^6^ Sciensano Brussels Belgium; ^7^ Department of Rehabilitation Sciences Ghent University Ghent Belgium; ^8^ Belgian Health Care Knowledge Centre (KCE) Brussels Belgium

**Keywords:** health services research, needs assessment, patient‐reported outcomes, psychotic disorders, questionnaire adaptation, schizophrenia spectrum disorders

## Abstract

**Background:**

The NEED (Needs Examination, Evaluation and Dissemination) framework provides a structured, multidimensional approach to assess unmet health‐related needs at both patient and societal levels, using predefined criteria, indicators and standardised data collection tools (e.g., questionnaire and interview guide). This paper outlines the methodological adaptations and considerations required when applying the NEED framework to psychotic disorders (PD). PD encompass a spectrum of conditions, including schizophrenia and schizoaffective disorder, and are characterised by overlapping symptom clusters and inconsistencies in communication of diagnoses in patient contact, which complicates research design and the delineation of study populations.

**Methods:**

In preparation for a NEED study in PD, a literature review and expert consultations informed the inclusion and exclusion criteria, choice of diagnostic terminology, refinement of data collection tools and the development of a recruitment strategy. The adapted tools were subsequently piloted in four interviews to assess clarity and feasibility.

**Results:**

Individuals with PD face distinct challenges for research participation. Stigma surrounding diagnostic labels contributes to a gap between clinical diagnosis and self‐identification, while limited illness insight, cognitive impairments (e.g., reduced attention span), and distrust of research further complicate participation. As the NEED methodology relies on self‐identification and self‐report, these characteristics required several adaptations. First, inclusion criteria were broadened from the initial intention of focusing on schizophrenia to PD to accommodate diagnostic uncertainty and variation in illness insight, while non‐stigmatising terminology was adopted for recruitment materials and survey instruments. Second, the questionnaire and interview guide were adapted to account for diagnostic variability, risk of non‐differential misclassification and the need for clear, sensitive wording. Third, the recruitment strategy incorporated materials that were as minimally stigmatising as possible, a wide range of actors and channels, on‐site support by researchers, and the option for proxy completion by legal representatives to enable inclusion of individuals with severe impairments.

**Discussion:**

This study highlights methodological challenges specific to conducting needs assessments in PD and describes the adaptations required to refine the NEED toolbox for this population. The reflections presented here aim to guide future applications of the NEED framework in mental health and other complex, stigmatised conditions.

**Patient or Public Contribution:**

A person with lived experience of a psychosis disorder was actively involved throughout the entire study design. Their input was crucial in shaping the study design, informing the relevance and formulation of the questions in the questionnaire, and ensuring the study reflected the perspectives and priorities of people directly affected by PD.

## 1 Introduction

The Belgian Health Care Knowledge Centre (KCE) and Sciensano have developed a multi‐dimensional framework, the NEED (needs examination, evaluation and dissemination) framework, to systematically assess unmet health‐related needs within a specific condition, which has recently been validated by experts [1, 2]. The primary objective of the NEED framework is not limited to measurement at the individual level, but to generate structured, comparable evidence on unmet needs across disease areas. These data are synthesised and disseminated, for example, through publicly available reports and the NEED database, with the aim of informing policy and decision‐making in, but not limited to, research prioritisation, regulatory assessment and reimbursement processes.

The framework integrates patient, societal and future perspectives and includes equity as a transversal component. The framework distinguishes between health needs (e.g., symptom burden), healthcare needs (e.g., access to appropriate services) and social needs (e.g., support in daily living or impact on work). The NEED framework includes 26 criteria and 46 indicators for assessing unmet health‐related needs [1, 2, 3]. Evidence for these criteria is collected using complementary methods, including literature review, database analysis, expert consultation, a patient (or proxy) questionnaire and patient (or proxy) interviews.

Based on an official call for proposals and subsequent expert prioritisation, schizophrenia was selected as one of the conditions for which a full NEED assessment would be conducted. The burden of schizophrenia alone has been increasing over recent decades, with an increase in the number of disability‐adjusted life years (DALYs) of 65% between 1990 and 2019 [4]. In the Belgian context, the prevalence of schizophrenia is approximately 0.4% with a burden of 279 per 100,000 DALYs [5]. During the design phase of the study, the scope was broadened to the overarching category of psychotic disorders (PD), which encompass severe mental health conditions affecting approximately 0.7% of the global population [6]. This category includes schizophrenia, schizoaffective disorder and other non‐affective PD. Individuals with PD represent a heterogeneous group, often characterised by stigma, limited illness insight, distrust in research and healthcare, and cognitive impairments such as reduced attention span [7]. These characteristics pose distinct challenges for the application of the NEED methodology [8, 9].

Within the NEED initiative, a set of generic tools to collect data on unmet health‐related needs have been developed, including a patient (or proxy) questionnaire and interview guide [10, 11]. The guidance for using these tools prescribes an adaptation to the health condition that is being studied [1]. Data collection by means of the NEED survey and patient interviews in the context of PD requires a tailored recruitment strategy, cognitively and linguistically sensitive instruments, and efforts to build participant trust [8, 12]. Carlsson et al. emphasise that despite the recognised need for qualitative research in this population, methodological aspects are often insufficiently reported [8]. This lack of transparency can undermine the trustworthiness and transferability of findings, highlighting the importance of methodological rigour and clarity in mental health research [8].

The development and methodological foundations of the generic NEED framework have been described in previous reports. In past case studies, the framework has been applied to three health conditions: (i) Crohn's disease, (ii) malignant melanoma and (iii) long COVID. In the present study, this framework was adapted to the context of PD, with modifications focused on contextual relevance and comprehensibility rather than the creation of a new measurement tool. Therefore, the aim of this paper is to present methodological considerations that arose during the design phase of adapting the NEED methodology to PD in Belgium. These reflections concern three main aspects: (i) setting inclusion and exclusion criteria, (ii) the adaptation of data collection instruments, including the questionnaire and interview guide, and (iii) the development of a feasible recruitment strategy. While some of the methodological adaptations described are specific to this health condition, others may be transferable to needs assessments in different disease areas. By providing transparency on the rationale underlying key methodological choices, this paper seeks to inform and support future applications of the NEED framework.

## 2 Methods

This paper describes the design phase of applying the NEED framework to PD, focusing on the key challenges encountered, mitigation strategies and resulting design choices. The NEED framework was previously developed and piloted in other disease areas, and this study represents its first application to PD, with a particular focus on condition‐ and context‐specific challenges. The paper reports on the resulting methodological design of the study rather than outcomes of its empirical application.

This study was centrally approved by the ethics committee of by the ethics committee of the ‘Erasmus Hospital Brussels’ (Ref. P2024/592).

### 2.1 Inclusion Criteria and Participant Definition

In line with recommendations for participatory research, the design phase of this study involved a broad research team including a patient (representative) from UilenSpiegel (i.e., a patient organisation for PD in Flanders, Belgium), psychiatrists (*n* = 2), clinical psychologists (*n* = 3) and other researchers (*n* = 11) [9]. This multidisciplinary input ensured that methodological adaptations of the NEED tools reflected both clinical expertise and lived experience. In particular, the design of inclusion criteria was informed by multidisciplinary group discussions, allowing for the integration of different perspectives, including considerations related to stigma and heterogeneity within the PD population.

### 2.2 Adaptation of the NEED Data Collection Tools

#### 2.2.1 The Generic NEED Patient Questionnaire and Interview Guide

The NEED toolbox includes, among others, a generic patient (or proxy) questionnaire and semi‐structured interview guide [10, 11]. The questionnaire captures patients’ perspectives on their general health status and various health‐related needs through a combination of structured response options and open‐ended questions, allowing both comparison across participants and qualitative elaboration. Rather than representing a de novo instrument, the NEED framework integrates items from existing validated tools. These include generic health status measures such as the *EQ‐5D‐5L* [13], as well as items informed by established surveys and instruments, including *BELHEALTH, PaRIS, the Patient Assessment of Chronic Illness Care, the Care Assessment Tool, the National Cancer Patient Experience Survey, the Work Productivity and Activity Impairment Questionnaire, the Zarit Burden Interview, and the Caregiver Burden Inventory*. Items were adapted and operationalised to fit the objectives of the NEED framework, while avoiding verbatim reproduction of copyrighted materials. A detailed description of the development process, including item selection and adaptation, is available in KCE Report 407C [11]. Nevertheless, when applying the framework in a new health condition, these tools prescribe an adaptation to the health condition that is being studied [10].

At the end of the survey, participants can express interest in taking part in a follow‐up interview. From those who indicate willingness, researchers select a diverse sample based on predefined segmentation criteria, including gender, time since diagnosis (categorised based on distribution in the sample), and living situation (distinguishing between individuals in residential care or long‐term hospital settings and those living at home), identified as key dimensions capturing variation in disease trajectory, care context, and daily functioning. Selected participants are re‐contacted to confirm their availability and receive further details about the interview. This targeted approach ensures sufficient representation of key subgroups. Stratification is employed to capture a wide range of experiences and perspectives across the PD population.

#### 2.2.2 Tailoring the Generic Data Collection Tools to PD

**Figure 1:**
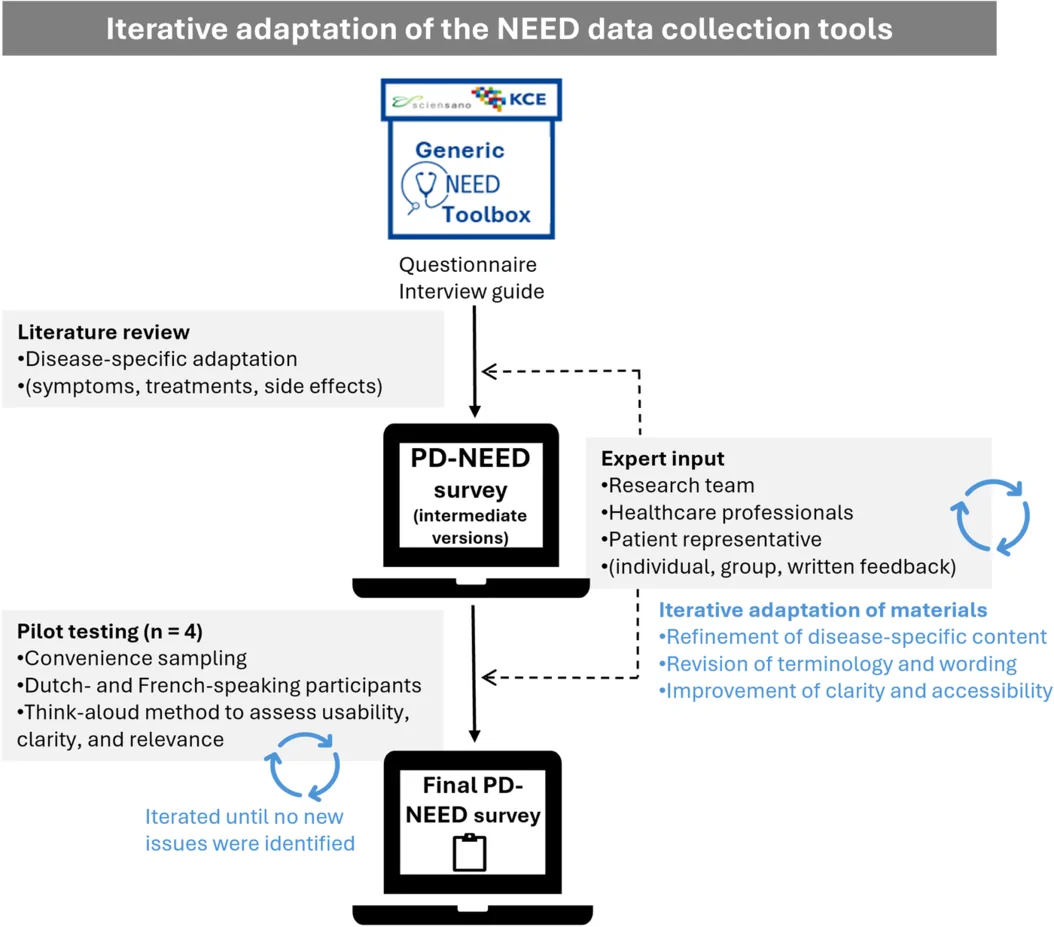
Adaptation process of the NEED data collection tool. The adaptation process followed an iterative and integrated approach, combining insights from literature review, expert input and pilot testing. Refinements were made through successive cycles rather than sequential steps. PD, psychotic disorder.

Adjustments to the generic NEED patient questionnaire were undertaken through an iterative and integrated adaptation process to ensure suitability for PD (Figure 1). This process combined insights from a literature review, expert input and pilot testing, which informed successive refinements of the questionnaire rather than being applied as strictly sequential steps.

The literature review informed the initial adaptation of the generic NEED questionnaire to the PD context, particularly for disease‐specific sections such as symptoms, treatments and side effects. In parallel, iterative feedback was obtained from the research team, two healthcare professionals, and a patient representative with lived experience. This input was collected through individual discussions, group discussions, and written feedback on the materials, and informed revisions aimed at improving clarity, relevance, and accessibility of both disease‐specific and generic questions.

The final questionnaire was implemented in LimeSurvey (Version 6.15.9), with technical checks to ensure functionality. Pilot testing was conducted with four participants (two Dutch‐speaking and two French‐speaking) who were recruited using convenience sampling based on their experience with PD and their ability to provide feedback on the questionnaire. All pilot interviewees were individuals with active experience with PD and one was a proxy for an individual with PD. All materials were developed in both Dutch and French. Translations were iteratively refined through input from the research team and a patient representative, and were assessed during pilot testing using a think‐aloud approach. The think‐aloud method was used to assess usability, clarity and relevance of the questions and to ensure clarity and comprehensibility. No formal back‐translation procedure was applied. Pilot testing was conducted iteratively, with sessions continued until no substantially new usability or comprehension issues were identified. Feedback from these sessions, along with input from clinical experts and a patient representative, informed further refinements to content and presentation.

The adapted questionnaire is provided in Supporting Information S2: Appendix II in its original Dutch and French versions, as used in the study and reflecting the importance of context‐specific phrasing. To support transparency and facilitate interpretation, readers are referred to the published NEED protocol, which includes the generic English version of the questionnaire that formed the basis for these adaptations [10].

### 2.3 Development of the Recruitment Strategy

For the development of recruitment materials, particular attention was given to the use of non‐stigmatising and patient‐sensitive language. Draft materials were discussed within the research team and reviewed by the patient representative to ensure appropriateness and clarity.

The recruitment strategy was initially based on the general approach outlined in the NEED protocol, including dissemination via online channels and physical flyers. This approach was further refined through group discussions within the multidisciplinary team, during which key actors, channels and strategies to reach diverse subgroups within the PD population were identified.

The final recruitment strategy was iteratively refined and validated with input from the patient representative to ensure relevance and accessibility.

Given the iterative nature of the adaptation process, the resulting adjustments are presented according to key areas of design, namely (i) inclusion criteria, (ii) adaptation of data collection tools and (iii) recruitment strategy.

## 3 Results

### 3.1 Setting Inclusion and Exclusion Criteria

Before adapting the data collection tools, it was necessary to define the target population and establish clear inclusion and exclusion criteria. The methodology of this study relied on voluntary participation and self‐identification, meaning that individuals needed to recognise themselves within the described target population and independently decide to participate. This, in turn, required a minimum level of illness insight, as reflecting on one's experiences and responding meaningfully to survey questions would otherwise be difficult.

Within the broader NEED framework, conditions are selected for assessment based on public submissions, which are subsequently rated by clinical experts against predefined criteria. Conditions with the highest scores are prioritised for full evaluation. Schizophrenia emerged as one of the conditions selected for further study. However, psychiatric diagnoses, including PD, pose unique challenges. They are based on symptom clusters rather than biologically defined disease mechanisms, which results in diagnostic uncertainty. PD are further characterised by diagnostic instability, as diagnoses may shift over time due to overlapping symptom profiles, especially in the early phases. Distinctions between conditions such as schizophrenia, schizoaffective disorder and psychotic depression is determined by subtle clinical nuances, for example, the temporal relationship between mood and psychotic symptoms [6]. Moreover, limited illness insight and the stigma associated with diagnostic labels such as *schizophrenia* may lead some individuals to resist or reject their diagnosis altogether, and may result in clinicians preferring less stigmatising diagnostic labels such as the more general term ‘psychosis’ or ‘psychotic disorder’.

These challenges complicate the delineation of inclusion and exclusion criteria. While precise diagnostic definitions are important for scientific validity, their translation into practical recruitment procedures is challenging. Diagnoses may be provisional, vary across clinical settings, or not be communicated to patients [6].

For the purposes of this study, the target population was defined as adults (≥ 18 years) residing in Belgium with a diagnosis of PD. These criteria informed both the development of the data collection instruments and the recruitment strategy, as discussed in the following sections.

#### 3.1.1 Terminology

An important consideration in defining the target population was the choice of terminology to describe the included diagnoses. The terminology needed to balance scientific precision, sensitivity to stigma, and contextual appropriateness. Certain diagnoses carry different levels of stigma. In particular, *schizophrenia* is strongly stigmatised, which may discourage individuals from identifying with the diagnosis and thus complicate recruitment [14]. In Belgium, as in many European countries, the term *schizophrenia* is frequently avoided in direct communication with patients due to its negative connotations [15, 16]. Consequently, many individuals do not self‐identify with this label, even if it has been used in their clinical record.

**Figure 2:**
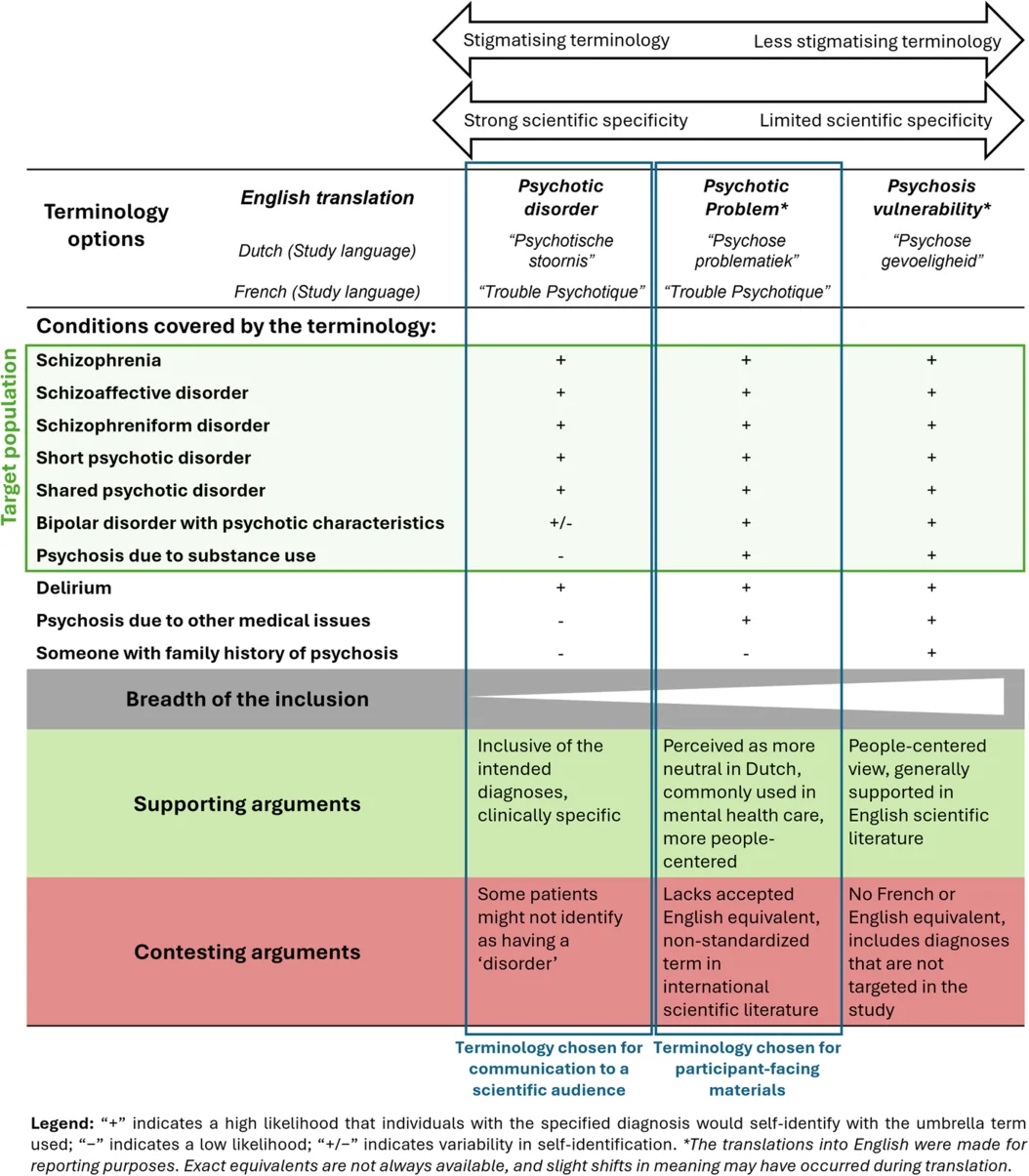
Comparative analysis of terminology options for identifying study populations with psychotic experiences. Each term is evaluated for scientific precision, stigma sensitivity and contextual appropriateness, highlighting choices for scientific and participant‐facing materials.

Several alternative terms were therefore evaluated, each varying in specificity and stigma sensitivity (Figure 2). Pilot interviews further highlighted linguistic and semantic challenges, especially given the need to provide materials in both Dutch and French. In Dutch, participants indicated that *psychosegevoeligheid* ('psychosis vulnerability') was perceived as the least stigmatising term. However, this concept does not translate well into French or English. In these languages, the terms *trouble psychotique* (French) and *psychotic disorder* (English) are generally recognised by both the scientific community and by patients and clinicians, and were therefore deemed most appropriate for the study.

The use of such non‐specific terminology, however, introduced another methodological challenge: it risked including individuals with transient, non‐recurrent psychotic experiences (e.g., illness‐induced psychosis), which diverges from the intended focus on recurrent or chronic psychotic conditions [17]. The final choice of terminology thus illustrates the broader tension between ensuring scientific and operational clarity while adopting language that facilitates self‐identification and aligns with the lived experiences of individuals affected by psychotic problems.

### 3.2 Adaptation of the Data Collection Tools

Table 1 provides an overview of the criteria and indicators included in the NEED assessment and the respective design considerations and adjustments. The final questionnaire consisted of 77 questions organised across 16 themes.

**Table 1:** Overview of criteria and indicators included in the NEED assessment.

Domain	Criteria	Indicator	Adjustments to the patient questionnaire^a^				Design consideration	Identified challenge	Mitigation strategy
			Copied^1^	Completed^2^	Adjusted^3^	NA^4^			
General	Health problem				✓		Terminology & clarity Terminology & clarity	Dissociation between persons’ experiences and diagnostic label provided by healthcare provider A psychotic disorder is not considered a disease, nor experienced by all individuals as one. This reference can be stigmatising and sensitive^b^	Question rephrased from 'what diagnosis do you have?' to 'what diagnosis did you get?' Any reference to 'disease' or 'illness' was replaced by “psychotic problem”^b^
Health	Impact on general health‐related quality of life	EQ‐5D‐5L average score			✓		Non‐differential misclassification Terminology & clarity	Potential first symptoms unclear. Questions E1‐E12 (Supporting Information S2: Appendix II) were perceived as not particularly relevant or even offending and unnecessary.	Brief explanation of potential first symptoms added, in case individuals are not able to remember. Explanation on why these questions are asked was included in the beginning of the survey as well as above this specific section.
	Impact on physical health	Experienced burden of physical symptoms		✓			Terminology & clarity	Dissociation between an individual's perception and the external world's view of reality.	Questions about 'experiencing things that did not exist' were rephrased as 'experiencing things that others do not' to avoid assumptions about what is real.
		Pain/discomfort today vs before	✓				Changing symptomatology Changing symptomatology Terminology & clarity	The onset of psychotic disorders is gradual, making it difficult to define a clear 'before onset of the symptoms' and 'today' Symptom severity fluctuates over time, so responses may not reflect the person's typical state and can vary depending on their condition the day of the survey. Effective medication may mask symptoms, resulting in little perceived difference between past and present. Questions E1‐E12 (Supporting Information S2: Appendix II) were perceived as not particularly relevant or even harsh and unnecessary.	Questions simplified and focused on recent experiences to improve interpretability. Aiming for a large sample to average out variability and planned subgroup analyses contrasting stable versus more symptomatic participants. Explanation on why these questions are asked was included in the beginning of the survey as well as above this specific section.
	Impact on psychological health	Experienced burden of psychological symptoms		✓					
		Anxiety/depression today vs before	✓						
	Impact on autonomy	Mobility today vs before onset of disease	✓						
		Self‐care today vs before onset of disease	✓						
		Usual activities before vs today	✓						
	Impact on life expectancy	Years of life lost (per death)				✓			
Healthcare	Effectiveness of treatment	Effectiveness of current treatment				✓			
	Burden of treatment	Experienced burden of treatment side‐effects		✓					
		Other experienced burden of treatment	✓						
	Quality of care	Experience with the organisation of care	✓						
		Quantity of information received	✓						
		Clarity of information received	✓						
		Involvement in treatment choice	✓						
		Diagnostic timing			✓		Non‐differential misclassification	As the survey targets individuals with an established diagnosis, including a response for those without a formal diagnosis would conflict with the inclusion criteria. A timeframe of less than 2 months may be too short for symptoms to fully manifest or for a reliable diagnosis to be established.	The answer options 'less than 2 months' and 'I have not received a diagnosis by a doctor' were excluded. To avoid overly fragmented response categories and better reflect clinical reality, the broader time frame 'less than 6 months' was used instead.
	Accessibility of care	Treatment availability				✓			
		Forgone care	✓						
Social	Impact on social life	Social support needs	✓						
	Impact on education	Impact on education	✓						
	Impact on work	Changed in working time	✓						
	Financial consequences	Perceived financial difficulties related to the health condition	✓						

*Note:* The table presents the criteria and indicators assessed in this study, based on the generic NEED patient questionnaire published by Maertens de Noordhout et al.Category 1—Copied: Question copied verbatim from the generic NEED questionnaire.

Category 2—Completed: Question completed as instructed in the generic template of the NEED questionnaire (e.g., disease‐specific symptoms, treatments or side effects).

Category 3—Adjusted: Question adapted in wording or content to better fit the psychotic disorder context.

Category 4—Not applicable: The indicator was not measured by the patient questionnaire but via expert opinions and/or database analysis.

^a^ Each item is marked to reflect the specific action taken during adaptation of the patient questionnaire.

^b^ This adjustment was applied throughout the whole questionnaire.

#### 3.2.1 Non‐Differential Misclassification

Participants are required to reflect on their disease trajectory from diagnosis to the present, necessitating recall of prior experiences related to illness onset and diagnosis. Given the gradual and heterogeneous emergence of psychotic symptoms, pilot participants found it challenging to pinpoint the onset of their condition. To address this, the questionnaire provided a brief explanation of what constitutes first psychotic symptoms, focusing on clearly identifiable features such as hallucinations, delusions and disorganised thinking, rather than non‐specific early signs (e.g., social withdrawal, reduced motivation).

Despite this clarification, pilot participants reported that questions about illness onset and diagnosis could be distressing or difficult to answer accurately, due to emotionally charged or blocked memories and difficulty recalling specific timelines (e.g., appearance of first symptoms, the timing of medical consultations, or the moment of diagnosis). The subtle and often unrecognised nature of symptoms further complicated retrospective reporting. This challenge could not be mitigated through design adjustments, as it reflects the intrinsic limitations of retrospective self‐reporting on sensitive experiences.

#### 3.2.2 Changing Symptomatology

Some pilot participants noted that some health‐related questions lacked temporal sensitivity and did not adequately capture the episodic course of PD. Questions about current health status failed to distinguish between periods of relapse and stability, limiting interpretability. Additionally, as PD are characterised by fluctuating symptom severity, responses may not reflect the individual's typical state and could vary depending on their condition at the time of survey completion. Several pilot participants explained that their symptoms were well‐controlled symptoms under medication, therefore perceived differences in symptoms and health state between prior to the onset of the symptoms and present were minimal.

To address this challenge, the questionnaire was revised to simplify wording and place greater emphasis on recent experiences. At the analytical level, the inclusion of a sufficiently large sample is expected to mitigate individual fluctuations, with variability in symptom course averaging out across respondents. In addition, subgroup analyses may help to account for heterogeneity, for example, by contrasting participants in relatively stable phases with those experiencing greater ongoing symptom burden.

#### 3.2.3 Non‐Stigmatising Terminology and Question Clarity

During pilot testing, several participants raised concerns about the use of potentially stigmatising or overly clinical terminology, including terms such as disease, disorder, or illness. These terms were therefore deleted. Similarly, expressions such as 'suffering from' were perceived by one participant as unnecessarily confronting and were avoided to prevent presupposing a uniformly negative experience.

To enhance clarity, additional explanations and concrete examples (e.g., of first symptoms) were incorporated for items that participants found difficult to interpret. Particular attention was paid to distinguishing between individuals’ lived experiences and the diagnostic labels assigned by healthcare professionals. Some participants did not identify with their clinical diagnosis but were able to report the diagnostic term used by their psychiatrist. Accordingly, the question on diagnosis was phrased in terms of ‘the diagnosis received from a psychiatrist’, rather than ‘the diagnosis you have’ to acknowledge possible discrepancies between self‐identification and medical labelling.

Further adaptations concerned items on care‐seeking behaviour. In the original version, these questions often presupposed voluntary action, whereas pilot participants reported that care had been initiated by others (e.g., family members, general practitioners, or emergency services). To better reflect diverse illness trajectories, such questions were reformulated. For instance, the item asking about ‘the moment the person decided to visit a physician’ was rephrased as ‘the first contact with a psychiatrist’ as this was often not the participant's own decision. Linguistic adjustments of this kind were implemented to ensure that the questionnaire more accurately captured participants’ perspectives and experiences.

### 3.3 Recruitment Strategy Design

The generic NEED toolbox relies on convenience sampling as the main recruitment method; in adapting this strategy to the context of PD careful attention was paid to the specific characteristics and challenges of the target population. The target population for this study is heterogeneous and difficult to delineate, which necessitated a broad and inclusive recruitment strategy. A proportion of individuals with PD may be institutionalised at the time of study, and these individuals are likely to experience more severe illness compared to those living independently. Ensuring their inclusion was essential to achieve a representative sample. However, recruitment within institutions poses specific ethical and practical challenges, and many institutionalised individuals are not always able to complete the survey autonomously.

#### 3.3.1 Recruitment Materials

The recruitment materials were designed to be accessible, engaging and emotionally considerate for the target population. Particular attention was given to the layout, colour palette, wording and visuals to ensure that the overall presentation was positive and inviting. Bright, uplifting colours and neutral or reassuring visuals were deliberately chosen to create a sense of openness and safety, while avoiding imagery or tones that could evoke distress. Wording and stigma adaptations were applied to the recruitment materials as mentioned under Section 2.3 and the term ‘trouble psychotique’ in French and ‘psychose problematiek’ were selected for patient‐facing materials for their neutrality and familiarity within the local mental health context, thereby fostering greater engagement and inclusivity. This approach aimed to reduce potential barriers to participation, promote understanding and create a welcoming invitation to take part in the study.

All recruitment materials are made available in Dutch and French to ensure accessibility across language regions. Once the recruitment message reaches potential participants, individuals are asked to self‐assess their eligibility based on the presented inclusion criteria. In line with recommendations from Iflaifel et al., participants may access the online questionnaire via the LimeSurvey platform or request an offline paper‐based version [12].

#### 3.3.2 Recruitment Actors and Channels

To address these challenges, the recruitment strategy prioritised representativeness by engaging a wide range of actors and channels. In light of the ongoing deinstitutionalisation of psychiatric care [18], outreach extended beyond psychiatric centres to also target individuals living in the community. Recruitment actors are divided into three main groups, each with tailored strategies.

**Figure 3:**
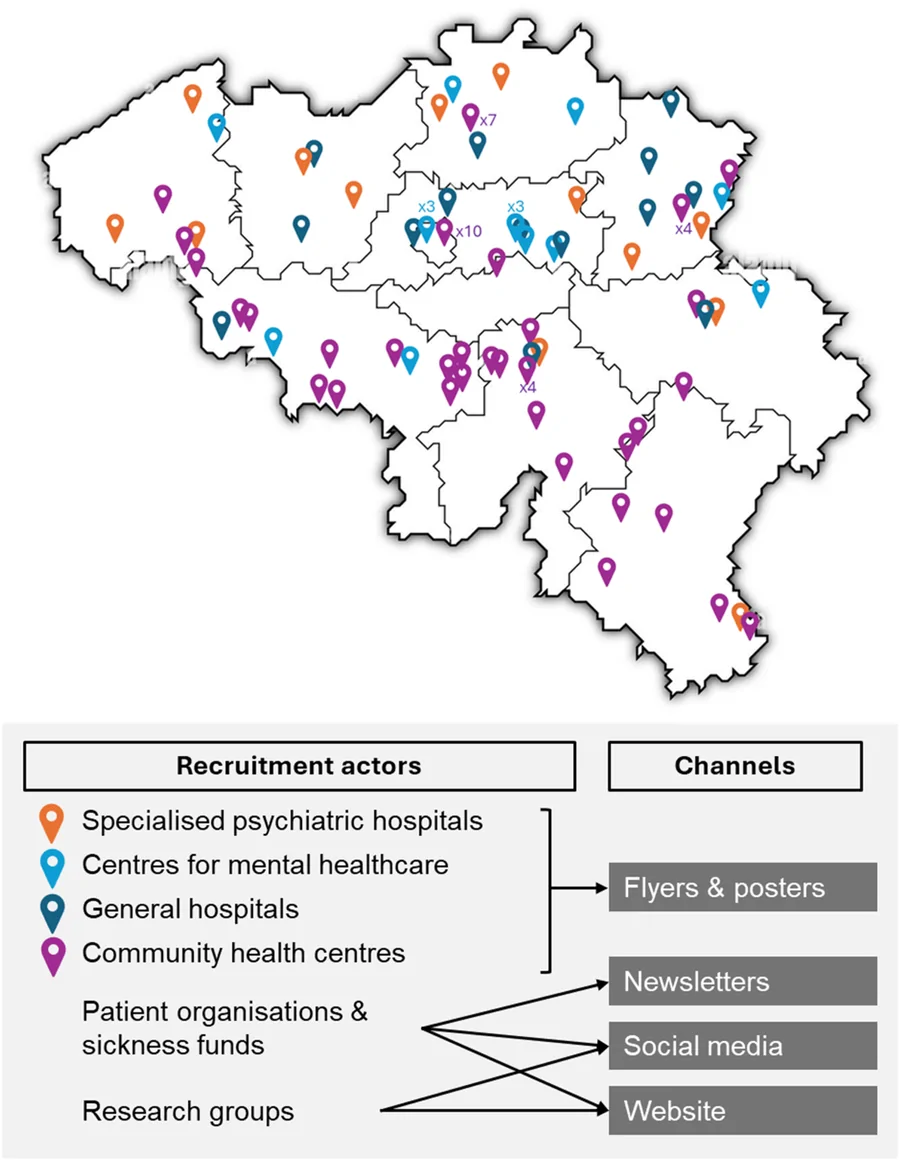
Geographical distribution of recruitment centres in Belgium. The pins indicate participating centres by region, ensuring balanced national coverage.

The first group includes community health centres, centres for mental healthcare, psychiatric hospitals, and general hospitals with psychiatric departments across Belgium. These institutions were asked to display posters and distribute flyers in waiting rooms. To ensure national representativeness, recruitment is geographically balanced, with an even distribution of participating centres across different regions of Belgium (Figure 3). The second group of recruitment actors consists of patient organisations and sickness funds, which are offered flexible options to disseminate recruitment materials via their newsletters, social media, websites, or a combination thereof. The choice of channels is left to each organisation, depending on their existing communication practices. The third group involves the research institutions participating in the study (e.g., KCE, Regulatory Sciences Research Group [KUL], Center for Psychiatry [KUL]), who will share the recruitment materials through their institutional websites and social media platforms.

#### 3.3.3 On‐Site Recruitment Support

To further improve accessibility, members of the research team visited 17 community health centres, nine centres for mental healthcare, and one general hospital in person, presenting the study to healthcare personnel for extra engagement and in specific centres (*n* = 7) also providing direct support to participants who would otherwise have been unable to complete the survey. This approach required significant time investment and the establishment of trust between researchers and participants. To facilitate this, researchers spent extended time on‐site, engaging informally with participants and healthcare staff, and allowing individuals to become familiar with their presence before inviting participation. In some settings, this included participating in routine activities or informal interactions, which helped lower barriers to engagement and foster a sense of safety and trust.

#### 3.3.4 Logistical and Ethical Considerations

Logistical and ethical considerations shaped the study's recruitment strategy. While inclusivity was prioritised, practical constraints limited outreach to certain groups, such as homeless individuals and people with substance use disorders, who have higher rates of PD but are difficult to reach without significant additional resources [19, 20, 21]. The initial study design also aimed to include adolescents aged 16–18 years; however, the ethical committee expressed reservations regarding the appropriateness of the proposed approach and the specific needs of this age group. Following their recommendation, adolescents were not included in the final recruitment.

For displaying posters and flyers in centres, separate local ethical clearance was required despite central ethical clearance. The research team actively contacted multiple centres and initiated local ethical approval procedures where required. However, in some cases, approval processes exceeded the study timeline, which limited inclusion of these centres. As a result, not all initially targeted sites could be included in the final recruitment strategy.

#### 3.3.5 Inclusion of Legal Representatives

To address accessibility, legal representatives were allowed to complete the survey for participants unable to do so themselves, ensuring inclusion of individuals with severe impairments or limited insight [17].

## 4 Discussion

The study aimed to tailor the generic NEED tools, including the patient (or proxy) questionnaire and interview guide for application in PD. Therefore, it was adapted through an iterative and integrated process, combining a literature review, expert discussions and pilot testing. Insights from these activities were continuously triangulated to inform successive refinements of the instrument. This is consistent with prior literature emphasising the need for sensitive and condition‐specific adaptations in mental health survey design [8]. Carlsson et al. have highlighted the frequent lack of transparency in qualitative research with people with severe mental illness, particularly regarding recruitment and ethical processes [8]. The present study addresses this gap by clearly documenting methodological considerations, including changes to the questionnaire design and decisions in the recruitment strategy.

This paper highlights the most important methodological challenges and study design considerations for the application of the NEED assessment framework in patients with PD, regarding (i) population selection, (ii) questionnaire design and (iii) recruitment strategy. It highlights the steps and adjustments made to tailor the generic NEED questionnaire to the disease‐specific context and the recruitment strategy applied to reach a representative sample in Belgium. The methodological challenges and mitigation strategies described in this paper reflect both the complexity of the target population and the challenge for methodological rigour in mental health research. A central methodological finding of this study is the challenge of balancing scientific rigour and meaningfulness of results with sensitivity to participants’ feelings and perceived stigma. Achieving both robust, generalisable data and a respectful, inclusive participant experience proved difficult to reconcile. Similarly, there was a need to weigh inclusivity and accessibility against ethical and logistical feasibility, as efforts to reach all relevant subgroups were constrained by practical and ethical considerations.

The findings of this study contribute to the ongoing debate on terminology and stigma in mental health research, particularly concerning PD. A systematic review highlights the absence of universally accepted definitions across the psychosis spectrum, underscoring the need for standardisation to advance both research and clinical care [22]. Increasing evidence shows that diagnostic labels such as schizophrenia can be stigmatising and carry fatalistic connotations, evoking associations with chronicity and dangerousness [15, 23, 24]. Efforts to rename or reframe schizophrenia, for instance, Japan's 2002 transition from ‘mind‐split disease’ to ‘integration disorder’, have improved clinical communication, patient acceptance and willingness to engage in treatment, though effects are often amplified when accompanied by broader anti‐stigma initiatives [15, 16, 25]. These findings demonstrate that terminology can shape how individuals understand and relate to their illness, influencing self‐stigma, recovery orientation and treatment engagement.

However, the evidence also suggests that renaming alone is insufficient to eliminate stigma, which is deeply embedded in cultural and structural factors [26]. Language in research and clinical contexts, therefore, functions not only as a diagnostic label but as a determinant of patient identity and social perception. Addressing these challenges requires comprehensive approaches that combine culturally sensitive terminology with structural reforms and public education.

Methodologically, this study highlights the importance of continuously refining patient‐centred assessment tools for mental health research. Conducting surveys or interviews with individuals with PD requires addressing fluctuating illness insight, cognitive limitations and stigma‐related barriers to participation [7]. Despite the identified challenges, a survey is generally considered a good method to assess health‐related needs of individuals' mental health conditions [27, 28, 29]. These challenges necessitate additional resources to support trust‐based, often in‐person recruitment strategies while ensuring inclusion beyond institutional settings. Extending recruitment to outpatient and community environments is particularly crucial, as many individuals with PD live outside formal care systems [18]. Sustainable progress in this area demands greater coordination and investment in mental health research infrastructure to reach underrepresented groups and to ensure studies are implemented with sufficient time and staff capacity [30].

From a policy perspective, these methodological reflections reinforce the need for continued structural and societal efforts to reduce stigma and promote equitable inclusion of people with PD in both care and research. Persistent stigma remains a major barrier to help‐seeking, adherence and social reintegration [7, 31, 32]. Strengthening anti‐stigma education, fostering recovery‐oriented care and developing international best‐practice guidance for research in severe mental illness would enhance both methodological rigour and the translation of evidence into more inclusive mental health systems.

### 4.1 Strengths and Limitations

A key strength during the adaptation of the NEED methods is the multidisciplinary composition of the research team, involving clinical and methodological experts as well as a patient representative, and patients during the pilot. Recognising that patients and healthcare professionals may have differing perspectives, the active participation of a patient co‐researcher throughout the study design (especially in shaping survey language, question scope and pilot testing) helped reduce cognitive burden and potential response error [33]. This aligns with best practices for developing self‐report tools for populations with cognitive vulnerabilities [34]. The involvement of a patient representative, affiliated with a patient organisation and with dual lived experience as both a person with psychosis and a caregiver, contributed valuable insights and helped foster trust with participants. The process also highlights the importance of flexibility and sensitive communication when collaborating with patient co‐researchers, given the potential for fluctuating health and emotional challenges. These considerations should be integrated into future mental health research involving patient collaborators.

This study has several methodological limitations. First, the scope was broadened from schizophrenia to PD to enhance inclusivity, which introduced heterogeneity and the potential participation of individuals not fully meeting inclusion criteria. This broader sampling frame may also affect the statistical power of subgroup analyses. Second, convenience sampling enabled wide outreach but may have introduced selection bias, particularly underrepresenting institutionalised or homeless individuals; these gaps will be further explored through planned subgroup analyses. Third, only four pilot interviews were conducted in a heterogeneous disease area, which may have limited the identification of all necessary adjustments, although thematic saturation was reached and participants provided valuable insights despite relatively high illness insight. Fourth, while in‐person recruitment in selected centres was planned to improve engagement, this approach proved resource‐intensive and dependent on site‐specific approvals, introducing potential participation bias linked to centre accessibility and researcher availability. Finally, as this study relied on self‐reported data collected at a single time point, recall bias and temporal variability in symptom severity may have influenced responses. Participants may best reflect their current state rather than their overall illness trajectory, limiting the ability to fully capture the fluctuating nature of PD and their long‐term impact on health‐related needs.

## 5 Conclusion

This paper reports key methodological challenges and resulting design choices in adapting the NEED framework to PD. The findings highlight three central areas requiring careful consideration: (i) defining inclusion criteria in the context of stigma and heterogeneity, (ii) ensuring patient‐sensitive terminology in all patient‐facing materials, and (iii) developing a broad and inclusive recruitment strategy. The results further demonstrate that iterative patient co‐creation is essential to ensure relevance, clarity, and acceptability of the adapted instrument. These findings provide practical methodological insights for future applications of the NEED framework, particularly in complex and vulnerable populations, by illustrating how common design challenges can be addressed in practice.

## Author Contributions

**Zilke Claessens:** investigation, writing – original draft, methodology, visualisation, writing – review and editing, data curation, formal analysis, conceptualisation. **Io Wens:** writing – review and editing, investigation, formal analysis, methodology. **Emma Olieslaeger:** formal analysis, investigation, writing – review and editing. **Ruud Van Winkel:** writing – review and editing, validation, supervision, funding acquisition, methodology. **Pierre Oswald:** validation, writing – review and editing, methodology. **Margaux Sageot:** writing – review and editing, validation, investigation. **Vicky Steyfkens:** validation, writing – review and editing, investigation. **Rani Claerman:** validation, writing – review and editing, methodology. **Robby De Pauw:** methodology, validation, writing – review and editing, funding acquisition, conceptualisation. **Laurence Kohn:** methodology, validation, writing – review and editing, conceptualisation. **Caroline Obyn:** methodology, validation, writing – review and editing. **Julie Paradis:** validation, writing – review and editing, methodology. **Claudia Schönborn:** methodology, validation, formal analysis, supervision, writing – review and editing, investigation, conceptualisation. **Irina Cleemput:** funding acquisition, supervision, validation, writing – review and editing, methodology, conceptualisation. **Charline Maertens Noordhout:** supervision, methodology, validation, writing – review and editing, funding acquisition, conceptualisation, project administration. **Isabelle Huys:** resources, supervision, validation, writing – review and editing, methodology, funding acquisition, project administration.

## Ethics Statement

Ethics approval was granted by the Ethics Committee Hospital‐Faculty Erasme‐ULB (P2024/592). The privacy and ethical team of KU Leuven (PRET) also approved the data management plan of the study (G‐2024‐8662). The study will be conducted in compliance with this protocol and guidelines for Good Clinical Practice. Personal data will be processed in accordance with the European General Data Protection Regulation (AVG/GDPR) 2016/679. Participants will provide informed consent, and all data will be pseudonymized to protect confidentiality. Special attention will be given to minimising participant burden and ensuring accessibility.

## Conflicts of Interest

The authors declare no conflicts of interest.

## Supporting information

Supporting File 1

Supporting File 2

## Data Availability

Data sharing not applicable to this article as no datasets were generated or analysed during the current study. The materials that support the findings of this study are available from the author, Charline Maertens de Noordhout, upon reasonable request. Results of the study will be presented and thoroughly discussed within the extensive research team, which includes members from the KCE, patient organisations, the Patient Preference Centre (KU Leuven), and clinicians from UZ Leuven. Findings will be disseminated via international peer‐reviewed journals, clinical and health economics conferences, and other academic platforms. Additionally, the results will be shared on the KCE website and the KU/UZ Leuven research unit websites. Most importantly, the data will be included in the publicly accessible NEED database, which features an interactive interface allowing users to search for specific needs within this patient population. A lay‐language summary of the results will also be prepared for distribution to patient organisations and recruiting parties to ensure accessibility to a broader audience.
