# Supplementary material for: Refining the NEED (Needs Examination, Evaluation and Dissemination) Methods for Use in Psychotic Disorders in Belgium: Lessons From the Design Phase
Source: Health Expect. 2026 Aug 31;29(5):e70724. doi: 10.1111/hex.70724 (PMC13527729; doi:10.1111/hex.70724)
Supplement: Supplementary file 1 [file HEX-29-e70724-s001.pdf]

Appendix II: Final Adapted Patient Questionnaire for the Psychotic Disorders NEED Study (Dutch and French Versions)

This appendix includes the final version of the adapted NEED patient survey, provided in Dutch (NL) and French (FR). These versions correspond to the data collection instruments discussed in the Methods section and were used in the field phase of the study.

NEED vragenlijst

ENQUÊTE OVER DE ONVERVULDE BEHOEFTEN VAN VOLWASSEN PATIËNTEN MET EEN PSYCHOTISCHE PROBLEMATIEK.

Hartelijk dank voor uw deelname aan ons onderzoek.

Ons onderzoek heeft als doel de behoeften van patiënten met een psychotische problematiek in kaart te brengen om hun zorg te verbeteren.

Deze vragenlijst is voor u als:

- U een psychotische problematiek hebt (bv. schizofrenie, schizoaffectieve stoornis, of een andere psychotische problematiek)
- U 18 jaar of ouder bent
- U in België woont (hoofdverblijfplaats)

Of

- U spreekt namens een volwassen persoon met een gediagnosticeerde psychotische problematiek, waarvan u de wettelijke vertegenwoordiger bent. Deze persoon woont in België en is niet in staat om voor zichzelf te spreken.

Dit onderzoek heeft als doel om de behoeften van patiënten met een psychotische problematiek te identificeren. Dit kan besluitmakers ondersteunen bij het nemen van beslissingen die de zorg kunnen verbeteren voor patiënten met een psychotische problematiek in het bijzonder en de maatschappij in het algemeen. Sommige vragen kunnen mogelijk minder goed aansluiten bij uw persoonlijke situatie of irrelevant aanvoelen. Deze vragen zijn van belang om de noden van patiënten uit andere ziektedomeinen met elkaar te vergelijken. Daarom verzoeken we u de vragen zo nauwkeurig mogelijk te beantwoorden, zodat we uw behoeften zo goed mogelijk kunnen capteren.

De resultaten van deze studie zullen begin 2026 gepubliceerd worden op de site van het KCE (www.kce.fgov.be) en op de site van NEED (www.health-needs.eu).

Voordat u akkoord gaat met deelname aan dit onderzoek, vragen wij u de volgende informatie over de vertrouwelijkheid van uw gegevens te lezen en toestemming te geven voor het gebruik van uw gegevens.

Er zijn 77 vragen in deze enquête.

Algemene informatie

U vult deze vragenlijst in *

Kies één van de volgende antwoorden
Kies één van de volgende mogelijkheden:

- Voor uzelf
- Voor een ander persoon waarvan u de wettelijke vertegenwoordiger bent (De persoon die u vertegenwoordigt is op dit moment niet in staat om de vragenlijst te beantwoorden. Door dit vakje aan te kruisen, neemt u deel aan dit onderzoek op basis van hun waarschijnlijke bereidheid om deel te nemen.)

Gelieve vanaf nu alle vragen te beantwoorden alsof u de persoon met de psychotische problematiek bent. Geef "Ik weet het niet" aan als u niet weet hoe de persoon zou reageren.

Beantwoord deze vraag alleen als aan de volgende voorwaarden is voldaan:
Antwoord was 'Voor een ander persoon waarvan u de wettelijke vertegenwoordiger bent (De persoon die u vertegenwoordigt is op dit moment niet in staat om de vragenlijst te beantwoorden. Door dit vakje aan te kruisen, neemt u deel aan dit onderzoek op basis van hun waarschijnlijke bereidheid om deel te nemen.) ' bij vraag '[D1]' (U vult deze vragenlijst in)

Met welk geslacht identificeert u zich? *

Kies één van de volgende antwoorden
Kies één van de volgende mogelijkheden:

- Man
- Vrouw
- Andere

In welk jaar bent u geboren? *

Uw antwoord mag minimaal 1920 zijn
In dit veld mag alleen een geheel getal worden ingevoerd.
Vul uw antwoord hier in:

-

Bedankt voor uw deelname.

Helaas voldoet u niet aan de voorwaarden om door te kunnen gaan met deze studie.

Beantwoord deze vraag alleen als aan de volgende voorwaarden is voldaan:
((D3.NAOK > 2007))

In welke provincie woont u? *

Beantwoord deze vraag alleen als aan de volgende voorwaarden is voldaan:
(((! is_empty(D3.NAOK) && (D3.NAOK <= 2007))))
Kies één van de volgende antwoorden
Kies één van de volgende mogelijkheden:

- Antwerpen
- Waals-Brabant
- Brussel
- Henegouwen
- Luik
- Limburg
- Luxemburg
- Namen
- Oost-Vlaanderen
- Vlaams-Brabant
- West-Vlaanderen
- Ik woon niet in België
- Ik weet het niet

Bedankt voor uw deelname.

Helaas voldoet u niet aan de voorwaarden om door te kunnen gaan met deze studie.

Beantwoord deze vraag alleen als aan de volgende voorwaarden is voldaan:
(((! is_empty(D3.NAOK) && (D3.NAOK <= 2007))) and (D4.NAOK == "AO12"))

Algemene informatie

Wat is het hoogste diploma of onderwijsniveau dat u tot nu toe hebt behaald? *

Kies één van de volgende antwoorden
Kies één van de volgende mogelijkheden:

- Geen diploma of basisonderwijs
- Lager secundair onderwijs
- Hoger secundair onderwijs
- Hoger onderwijs van het korte type (bv. bachelor)
- Hoger onderwijs van het lange type (bv. master)
- Doctoraat
- Ik weet het niet
- Overige

U woont.... *

Kies één van de volgende antwoorden
Kies één van de volgende mogelijkheden:

- Alleen
- Alleen met kinderen
- Samen (zonder kinderen)
- Samen (met kinderen)

- Met één of twee ouders
- In cohousing (gemeenschappelijk wonen)
- In een psychiatrisch verzorgingstehuis
- In beschut wonen
- In een andere begeleide woonvorm
- In het ziekenhuis (tijdelijk)
- In een woonzorgcentrum
- Ik weet het niet
- Overige

In welk land bent u geboren? *

Kies één van de volgende antwoorden
Kies één van de volgende mogelijkheden:

- België
- Ander land
- Ik weet het niet

Selecteer het betreffende land *

Beantwoord deze vraag alleen als aan de volgende voorwaarden is voldaan:
Antwoord was 'Ander land ' bij vraag ' [D7]' (In welk land bent u geboren?)
Kies één van de volgende antwoorden
Kies één van de volgende mogelijkheden:

- Afghanistan
- Zuid-Afrika
- Albanië
- Algerije
- Duitsland
- Andorra
- Angola
- Anguilla
- Antarctica
- Antigua en Barbuda
- Saoedi-Arabië
- Argentinë
- Armenië
- Aruba
- Australië
- Oostenrijk
- Azerbeidzjan
- Bahama's
- Bahrein
- Bangladesh
- Barbaos
- Belize
- Benin

- Bermuda
- Bhoutan
- Wit-Rusland
- Bolivia
- Bonaire
- Bosnië-Herzegovina
- Botswana
- Brazilië
- Brunei
- Bulgarije
- Burkina Faso
- Burundi
- Cambodja
- Kameroen
- Canada
- Kaapverdië
- Chili
- China
- Cyprus
- Colombia
- Comoren
- Congo (-Brazzaville)
- Congo (Democratische Republiek)
- Noord-Korea
- Zuid-Korea (Republiek Korea)
- Costa Rica
- Ivoorkust
- Kroatië
- Cuba
- Curaçao
- Denemarken en Faeröer
- Djibouti
- Dominica
- Egypte
- El Salvador
- Verenigde Arabische Emiraten
- Ecuador
- Eritrea
- Spanje en Canarische Eilanden
- Estland
- eSwatini (Swaziland)
- Verenigde Staten van Amerika
- Ethiopië
- Fiji
- Finlande
- Frankrijk
- Gabon
- Gambie
- Georgië

- Ghana
- Gibraltar
- Griekenland
- Grenada
- Groenland
- Guadeloupe
- Guam
- Guatemala
- Guinee-Bissau
- Guinee (Conakry)
- Equatoriaal-Guinea
- Guyana (Georgetown)
- Frans-Guyana
- Haïti
- Honduras
- Hong Kong
- Hongarije
- Kerstmis-eiland
- Kaaimaneilanden
- Cookeilanden
- Falklandeilanden
- Marshalleilanden
- Salomonseilanden
- Amerikaanse Maagdeneilanden
- Britse Maagdeneilanden
- India
- Indonesië
- Irak
- Iran
- Ierland
- IJsland
- Israël
- Italië
- Jamaica
- Japan
- Jordanië
- Kazachstan
- Kenia
- Kirgizië
- Kiribati
- Kosovo
- Koweït
- Laos
- Lesotho
- Letland
- Libanon
- Liberia
- Libië
- Liechtenstein

- Litouwen
- Luxemburg
- Macau (China)
- Noord-Macedonië
- Madagaskar
- Maleisië
- Malawi
- Maldiven
- Mali
- Malta
- Noordelijke Marianen (Amerikaans)
- Marokko
- Martinique
- Mauritius
- Mauritanië
- Mayotte
- Mexico
- Micronesië
- Moldavië
- Monaco
- Mongolië
- Montenegro
- Montserrat (Brits)
- Mozambique
- Myanmar
- Namibië
- Nauru
- Nepal
- Nicaragua
- Niger
- Nigeria
- Niue
- Noorwegen
- Nieuw-Caledonië
- Nieuw-Zeeland
- Oman
- Oeganda
- Oezbekistan
- Pakistan
- Palau
- Palestina
- Panama
- Papoea-Nieuw-Guinea
- Paraguay
- Nederland
- Peru
- Filipijnen
- Pitcairn
- Polen

- Frans-Polynesië
- Porto Rico
- Portugal, Azoren, Madeira
- Qatar
- Centraal-Afrikaanse Republiek
- Dominicaanse Republiek
- Tsjechië
- Réunion
- Roemenië
- Verenigd Koninkrijk
- Rusland
- Rwanda
- Westelijke Sahara
- Saint-Barthélemy
- Saint-Kitts en Nevis
- Sint-Eustatius en Saba
- San Marino
- Saint-Martin (Frankrijk)
- Sint Maarten (Nederland)
- Saint-Pierre en Miquelon
- Saint Vincent en de Grenadines
- Sint-Helena - Tristan Da Cunha - Ascension
- Saint Lucia
- Samoa
- Amerikaans Samoa
- Sao Tomé en Principe
- Senegal
- Servië
- Seychellen
- Sierra Leone
- Singapore
- Slowakije
- Slovenië
- Somalië
- Soedan
- Zuid-Soedan
- Sri Lanka
- Zweden
- Zwitserland
- Suriname
- Syrië
- Tadjikistan
- Taiwan
- Tanzania
- Tsjaad
- Thaïland
- Oost-Timor
- Togo
- Tokelau (Nieuw-Zeeland)

- Tonga
- Trinidad en Tobago
- Tunesië
- Turkmenistan
- Turks- en Caicoseilanden (Brits)
- Turkije
- Tuvalu
- Oekraïne
- Uruguay
- Vanuatu
- Venezuela
- Vietnam
- Wallis en Futuna
- Jemen
- Zambia
- Zimbabwe
- Ik weet het niet
- Overige

Wat was uw <u>voornaamste</u> beroepsstatuut vlak <u>voor het begin van uw psychotische problematiek</u>? *

Kies één van de volgende antwoorden
Kies één van de volgende mogelijkheden:

- Betaald werk (werknemer of zelfstandige)
- Werkzoekende, werkloos, ontvanger van een leefloon (OCMW)
- Huisvrouw of huisman, zonder uitkering
- Student
- Gepensioneerde
- Invaliditeit
- Ik weet het niet
- Overige

Heeft u betaald werk gedaan sinds het begin van de eerste symptomen van uw psychotische problematiek?

*****

Beantwoord deze vraag alleen als aan de volgende voorwaarden is voldaan:
Antwoord ONGELIJK 'Gepensioneerde' bij vraag ' [D9]' (Wat was uw voornaamste beroepsstatuut vlak voor het begin van uw psychotische problematiek?)
Kies één van de volgende antwoorden
Kies één van de volgende mogelijkheden:

- Ja
- Neen
- Ik weet het niet

Beschouw als "eerste symptomen van uw psychotische problematiek" de eerste verschijnselen van je aandoening, zoals het horen of zien van dingen waarvan anderen beweren dat ze er niet zijn, het hebben van gedachten die niet overeenkomen met de werkelijkheid die anderen waarnemen, of het idee hebben dat iemand je opzettelijk kwaad wil doen. Als je niet weet wanneer je symptomen begonnen, beschouw dan in plaats daarvan je eerste contact met psychiatrische zorg.

Wat is uw <u>voornaamste</u> beroepsstatuut <u>op dit moment</u>? *

Kies één van de volgende antwoorden
Kies één van de volgende mogelijkheden:

- Betaald werk (werknemer of zelfstandige)
- Werkzoekende, werkloos, ontvanger van een leefloon (OCMW)
- Huisvrouw of huisman, zonder uitkering
- Student
- Gepensioneerde
- Invaliditeit
- Ik weet het niet
- Overige

Rekening houdend met alle inkomsten van uw huishouden, in welke mate slaagt u erin om aan het einde van elke maand rond te komen? *

Kies één van de volgende antwoorden
Kies één van de volgende mogelijkheden:

- Zeer gemakkelijk
- Gemakkelijk
- Vrij gemakkelijk
- Met enige moeite
- Met moeite
- Met veel moeite
- Ik weet het niet

Met andere woorden, in welke mate zijn de inkomsten van uw huishouden voldoende om elke maand al uw uitgaven te dekken?

Met betrekking tot uw gezondheidsprobleem

Hoe lang geleden ongeveer kreeg u de diagnose van uw psychotische problematiek? *

Kies één van de volgende antwoorden
Kies één van de volgende mogelijkheden:

- Minder dan 6 maanden
- 6 maanden tot 2 jaar

- 2 tot 5 jaar
- 5 tot 10 jaar
- Meer dan 10 jaar
- Ik weet het niet

Geef bij benadering het aantal jaren aan: *

Beantwoord deze vraag alleen als aan de volgende voorwaarden is voldaan:
Antwoord was 'Meer dan 10 jaar ' bij vraag ' [D13]' (Hoe lang geleden ongeveer kreeg u de diagnose van uw psychotische problematiek?)
In dit veld mogen alleen cijfers ingevoerd worden.
Uw antwoord moet tussen 10 en 100 liggen
Vul uw antwoord hier in:

-

Hoeveel tijd zat er tussen de eerste symptomen van uw psychotische problematiek en het eerste contact met een psychiater? *

Kies één van de volgende antwoorden
Kies één van de volgende mogelijkheden:

- Minder dan 1 maand
- Tussen 1 en 6 maanden
- Tussen 6 maanden en 1 jaar
- Tussen 1 en 2 jaar
- Meer dan 2 jaar
- Ik weet het niet

Hoeveel tijd zat er tussen uw eerste medische consultatie voor uw psychotische problematiek en de diagnose? *

Kies één van de volgende antwoorden
Kies één van de volgende mogelijkheden:

- Minder dan 1 maand
- Tussen 1 en 6 maanden
- Tussen 6 maanden en 1 jaar
- Tussen 1 en 2 jaar
- Meer dan 2 jaar
- Ik weet het niet

Welke diagnose heeft u gekregen? *

Beantwoord deze vraag alleen als aan de volgende voorwaarden is voldaan:
((D13.NAOK != "A7"))
Alles selecteren wat van toepassing is
Kies alle voor u geldende mogelijkheden:

- Schizofrenie
- Schizoaffectieve stoornis
- Schizofreniforme stoornis
- Delirium
- Korte psychotische stoornis
- Gedeelde psychotische stoornis
- Psychotische stoornis door middelen gebruik
- Psychotische stoornis door een ander medisch probleem
- Niet gespecifieerde psychotische stoornis
- Bipolaire stoornis met psychotische kenmerken
- Ik weet het niet
- Ik wens hierop niet te antwoorden
- Overige:

Bedankt voor uw deelname.

Helaas voldoet u niet aan de voorwaarden om door te kunnen gaan met deze studie aangezien we een psychotische problematiek door een andere medische aandoening niet includeren.

Beantwoord deze vraag alleen als aan de volgende voorwaarden is voldaan:
((DSD1_A1.NAOK != "Y" and DSD1_A11.NAOK != "Y" and DSD1_A12.NAOK != "Y" and DSD1_A2.NAOK != "Y" and DSD1_A3.NAOK != "Y" and DSD1_A4.NAOK != "Y" and DSD1_A5.NAOK != "Y" and DSD1_A6.NAOK != "Y" and DSD1_A7.NAOK != "Y" and DSD1_A8.NAOK == "Y" and DSD1_A9.NAOK != "Y"))

Bent u ooit opgenomen geweest in het ziekenhuis wegens uw psychotische problematiek? Denk alleen aan verblijven met een overnachting in het ziekenhuis. *

Beantwoord deze vraag alleen als aan de volgende voorwaarden is voldaan:
((DSD1_A1.NAOK == "Y" or DSD1_A11.NAOK == "Y" or DSD1_A12.NAOK == "Y" or DSD1_A2.NAOK == "Y" or DSD1_A3.NAOK == "Y" or DSD1_A4.NAOK == "Y" or DSD1_A5.NAOK == "Y" or DSD1_A6.NAOK == "Y" or DSD1_A7.NAOK == "Y" or DSD1_A9.NAOK == "Y" or DSD1_A10.NAOK == "Y"))
Kies één van de volgende antwoorden
Kies één van de volgende mogelijkheden:

- Ja
- Neen
- Ik weet het niet

{if(((is_empty(DSD1_A1.NAOK)) and (is_empty(DSD1_A2.NAOK)) and (is_empty(DSD1_A3.NAOK)) and (is_empty(DSD1_A4.NAOK)) and (is_empty(DSD1_A5.NAOK)) and (is_empty(DSD1_A6.NAOK)) and (is_empty(DSD1_A7.NAOK)) and (is_empty(DSD1_A9.NAOK)) and (is_empty(DSD1_A11.NAOK)) and (is_empty(DSD1_A12.NAOK)) and (DSD1_A8.NAOK == "Y")),'uitgesloten','in')}

Andere gezondheidsproblemen

Hebt u last van gezondheidsproblemen die geassocieerd kunnen worden met uw psychotische problematiek? *

Kies het toepasselijke antwoord voor elk onderdeel:

	Ja	Neen	Ik weet het niet
Obsessieve-compulsieve stoornis (OCS)			
Depressie			
Bipolaire stoornis			
Alcoholprobleem			
Problematiek gerelateerd aan gebruik van drugs of andere verslavende middelen			
Angst- of paniekstoornis			
Post-traumatische stress			
Persoonlijkheidsstoornis			
Autisme spectrum stoornis (ASS)			
Aandachtsstoornis met of zonder hyperactiviteit (ADHD)			
Eetstoornis (bijv. anorexia, boulemia)			

Heeft u ook last van andere gezondheidsproblemen? *

Kies één van de volgende antwoorden
Kies één van de volgende mogelijkheden:

- Ja
- Neen
- Ik weet het niet

Van welke andere gezondheidsproblemen heeft u last? <u>U mag meer dan één vakje aankruisen</u>. Vermeld ook de precieze naam van uw gezondheidsprobleem of -problemen, indien gekend.

Als u de diagnose van uw andere gezondheidsprobleem (nog) niet weet, vink dan het vakje "Ander gezondheidsprobleem zonder gekende diagnose" aan.

*****

Beantwoord deze vraag alleen als aan de volgende voorwaarden is voldaan:

Antwoord was 'Ja' bij vraag ' [D14]' (Heeft u ook last van andere gezondheidsproblemen?)
Vul alleen een opmerking in als u een antwoord kiest.
Kies alle voor u geldende mogelijkheden en geef een toelichting:

- Hart- en vaataandoeningen (bv. hoge bloeddruk)
- Aandoeningen van het ademhalingsstelsel (bv. astma)
- Aandoeningen van het spijsverteringsstelsel (bv. prikkelbaredarmsyndqoom)
- Huidaandoeningen
- Aandoeningen van het bewegingsstelsel (beenderen, gewrichten of spieren) (bv. artrose, reumatoïde artritis)
- Andere mentale/psychische aandoeningen die niet vermeld werden in de vorige vraag
- Neurologische ontwikkelingsstoornis (bv. ontwikkelingsaandoeningen)
- Ziekten van het zenuwstelsel (neurologische ziekten) (bv. dementie, multiple sclerose)
- Hematologische (bloed) aandoeningen (bv. bloedarmoede)
- Immuunziektes (bv. allergieën, auto-inflammatoire ziekten)
- Endocriene, voedings- of stofwisselingsaandoeningen (bv. diabetes, hoge cholesterol, obesitas, onvoldoende werkende schildklier)
- Oogaandoeningen
- Ooraandoeningen of aandoeningen van het evenwichtsorgaan
- Aandoeningen aan het urine- (bv. blaas, nieren) en voortplantingsstelsel (bv. nierfalen, endometriose)
- Slaapstoornissen
- Aandoeningen die meerdere systemen aantasten (bv. psoriasis
- Ander gezondheidsprobleem zonder gekende diagnose
- Ik weet het niet
- Anders:

Uw algemene gezondheidstoestand

Sommige van de volgende vragen hebben mogelijk niet direct betrekking op uw situatie. Ze helpen ons echter wel om uw context te begrijpen. Hartelijk dank dat u de tijd neemt om deze vragen te beantwoorden.

We willen graag weten hoe u uw gezondheid ervaart. Kruis het vakje aan naast de verklaring die het beste uw gezondheid beschrijft net VOOR dat de eerste symptomen van uw psychotische problematiek zich voordeden.

Beschouw als "eerste symptomen van uw psychotische problematiek" de eerste verschijnselen van je aandoening, zoals het horen of zien van dingen waarvan anderen beweren dat ze er niet zijn, het hebben van gedachten die niet overeenkomen met de werkelijkheid die anderen waarnemen, of het idee hebben dat iemand je opzettelijk kwaad wil doen. Als je niet weet wanneer je symptomen begonnen, beschouw dan in plaats daarvan je eerste contact met psychiatrische zorg

Uw mobiliteit vóór de eerste symptomen van uw psychotische problematiek. *

Kies één van de volgende antwoorden
Kies één van de volgende mogelijkheden:

- Ik had geen problemen met rondwandelen
- Ik had een beetje problemen met rondwandelen
- Ik had matige problemen met rondwandelen
- Ik had ernstige problemen met rondwandelen
- Ik was niet in staat om rond te wandelen
- Ik weet het niet

Uw onafhankelijkheid vóór de eerste symptomen van uw psychotische problematiek. *

Kies één van de volgende antwoorden
Kies één van de volgende mogelijkheden:

- Ik had geen problemen om mijzelf te wassen of aan te kleden
- Ik had een beetje problemen met mijzelf te wassen of aan te kleden
- Ik had matige problemen met mijzelf te wassen of aan te kleden
- Ik had ernstige problemen met mijzelf te wassen of aan te kleden
- Ik was niet in staat mijzelf te wassen of aan te kleden
- Ik weet het niet

Uw dagelijkse activiteiten vóór de eerste symptomen van uw psychotische problematiek (bijv.: werk, studies, boodschappen doen, schoonmaken, openbaar vervoer nemen, gezinsactiviteiten, vrienden zien of hobby's) *

Kies één van de volgende antwoorden
Kies één van de volgende mogelijkheden:

- Ik had geen problemen met mijn dagelijkse activiteiten
- Ik had een beetje problemen met mijn dagelijkse activiteiten
- Ik had matige problemen mijn dagelijkse activiteiten
- Ik had ernstige problemen met mijn dagelijkse activiteiten
- Ik was niet in staat mijn dagelijkse activiteiten uit te voeren
- Ik weet het niet

Uw pijn of ongemak vóór de eerste symptomen van uw psychotische problematiek. *

Kies één van de volgende antwoorden
Kies één van de volgende mogelijkheden:

- Ik had geen pijn of ongemak
- Ik had een beetje pijn of ongemak
- Ik had matige pijn of ongemak
- Ik had ernstige pijn of ongemak

- Ik had extreme pijn of ongemak
- Ik weet het niet

Uw angst of depressie <u>vóór</u> de eerste symptomen van uw psychotische problematiek. *

Kies één van de volgende antwoorden
Kies één van de volgende mogelijkheden:

- Ik was niet angstig of depressief
- Ik was een beetje angstig of depressief
- Ik was matig angstig of depressief
- Ik was erg angstig of depressief
- Ik was extreem angstig of depressief
- Ik weet het niet

We willen graag weten hoe goed of slecht uw gezondheid was VÓÓR de eerste symptomen van uw psychotische problematiek op een schaal van 0 tot 100.

100 komt overeen met de <u>best denkbare</u> gezondheid.
0 komt overeen met de <u>slechtst denkbare</u> gezondheid.

Vul een getal in tussen 0 en 100 om uw gezondheidstoestand VÓÓR de eerste symptomen van uw psychotische problematiek weer te geven.

Uw antwoord moet tussen 0 en 100 liggen
In dit veld mag alleen een geheel getal worden ingevoerd.
Vul uw antwoord hier in:

-

Met de volgende vragen willen we graag weten hoe het met uw <u>huidige</u> gezondheid is gesteld. Kruis het vakje aan naast de verklaring die het beste beschrijft wat uw gezondheid <u>VANDAAG</u> is.

Uw mobiliteit vandaag *

Kies één van de volgende antwoorden
Kies één van de volgende mogelijkheden:

- Ik heb geen problemen met rondwandelen
- Ik heb een beetje problemen met rondwandelen
- Ik heb matige problemen met rondwandelen
- Ik heb ernstige problemen met rondwandelen
- Ik ben niet in staat om rond te wandelen
- Ik weet het niet

Uw autonomie vandaag *

Kies één van de volgende antwoorden
Kies één van de volgende mogelijkheden:

- Ik heb geen problemen met mijzelf te wassen of aan te kleden
- Ik heb een beetje problemen met mijzelf te wassen of aan te kleden
- Ik heb matige problemen met mijzelf te wassen of aan te kleden
- Ik heb ernstige problemen met mijzelf te wassen of aan te kleden
- Ik ben niet in staat mijzelf te wassen of aan te kleden
- Ik weet het niet

Uw huidige activiteiten (bijv. werk, studies, boodschappen doen, schoonmaken, openbaar vervoer nemen, gezinsactiviteiten, vrienden zien of hobby's) vandaag *

Kies één van de volgende antwoorden
Kies één van de volgende mogelijkheden:

- Ik heb geen problemen met mijn dagelijkse activiteiten.
- Ik heb kleine problemen met mijn dagelijkse activiteiten
- Ik heb matige problemen met mijn dagelijkse activiteiten
- Ik heb ernstige problemen met mijn dagelijkse activiteiten
- Ik ben niet in staat mijn dagelijkse activiteiten uit te voeren
- Ik weet het niet

Uw pijn of ongemak vandaag *

Kies één van de volgende antwoorden
Kies één van de volgende mogelijkheden:

- Er is geen pijn of ongemak
- Ik heb een beetje pijn of ongemak
- Ik heb matige pijn of ongemak
- Ik heb ernstige pijn of ongemak
- Ik heb extreme pijn of ongemak
- Ik weet het niet

Uw angst of depressie vandaag *

Kies één van de volgende antwoorden
Kies één van de volgende mogelijkheden:

- Ik ben niet angstig of depressief
- Ik ben een beetje angstig of depressief
- Ik ben matig angstig of depressief
- Ik ben erg angstig of depressief
- Ik ben extreem angstig of depressief
- Ik weet het niet

We willen graag weten hoe goed of slecht uw gezondheid VANDAAG is op een schaal van 0 tot 100.

100 komt overeen met de <u>beste gezondheid</u> die u zich kunt voorstellen.
0 komt overeen met de <u>slechtste gezondheid</u> die u zich kunt voorstellen.

Vul een getal in tussen 0 en 100 om uw gezondheidstoestand VANDAAG weer te geven.

Uw antwoord moet tussen 0 en 100 liggen
In dit veld mag alleen een geheel getal worden ingevoerd.
Vul uw antwoord hier in:

-

Behandelingen gerelateerd aan uw psychotische problematiek

Werd u <u>de afgelopen 5 jaar</u> behandeld voor uw psychotische problematiek? *

Kies één van de volgende antwoorden
Kies één van de volgende mogelijkheden:

- Ja
- Neen
- Ik weet het niet

Welke behandelingen heeft u <u>de afgelopen 5 jaar</u> gekregen voor uw psychotische problematiek?

*****

Beantwoord deze vraag alleen als aan de volgende voorwaarden is voldaan:
Antwoord was 'Ja' bij vraag ' [HC2]' (Werd u de afgelopen 5 jaar behandeld voor uw psychotische problematiek?)
Alles selecteren wat van toepassing is
Kies alle voor u geldende mogelijkheden:

- Atypische antipsychotica (‘tweede generatie’ antipsychotica) (bv. Aripiprazole, Abilify, Sycrest, Reagila, Olanzapine, Zypadhera, Invega, Xeplion, Trevicta, Quétiapine, Risperidone, Serdolect)
- Klassieke antipsychotica (‘eerste generatie’ antipsychotica) (bv. Clopixol, Haldol, Nozinan, Fluanxol, Etumine, Deanxit)
- Clozapine (bv. Clozapine Sandoz, Leponex)

- Antidepressiva gebruikt voor depressie, obsessief-compulsieve stoornis of paniekstoornis (bv. Citalopram, Escitalopram, Fluoxetine, Sertraline, Edronax, Redomex, Duloxetine, Venlafaxine)
- Kalmeringsmiddelen (bv. Diazepam, Valium, Lormetazepam, Lorazepam)
- Psychotherapie (bv. cognitieve gedragstherapie, psycho-educatie, etc.)
- Electroconvulsietherapie (ECT)
- Andere niet-invasieve hersenstimulatie: transcraniële gelijkstroomstimulatie (tDCS) of repetitieve transcraniële magnetische stimulatie (rTMS)
- Diepe hersenstimulatie
- Lichaamsbeweging
- Ondersteuning van anderen die dezelfde aandoening hebben
- Ik weet het niet
- Overige:

Welke bijwerking(en) heeft u tijdens of na de behandeling voor uw psychotische problematiek gehad in de afgelopen 5 jaar? *

Beantwoord deze vraag alleen als aan de volgende voorwaarden is voldaan:
((HC2.NAOK == "A1"))
Alles selecteren wat van toepassing is
Kies alle voor u geldende mogelijkheden:

- Gewichtstoename
- Hyperglycemie (te veel suiker in het bloed) of diabetes
- Hoge cholesterol (overtollige vetten in het bloed)
- Tekort aan witte bloedcellen
- Bewegingsstoornissen
- Rusteloosheid, moeite met stilzitten, behoefte om te bewegen
- Onvrijwillige en soms pijnlijke spiersamentrekkingen
- Beven en/of gezichtsstijfheid
- Onwillekeurige gezichtsbewegingen, zoals kauwen, tong uitsteken of knipperen
- Vermoeidheid of slaperigheid
- Duizeligheid
- Droge mond of ogen
- Misselijkheid
- Constipatie
- Myocarditis (ontsteking van de hartspier)
- Seksuele dysfunctie (bv. verminderd libido)
- Geheugenproblemen of "mentale mist"
- Emotionele afvlakking
- Geen nadelige effecten
- Overige:

Geef aan in welke mate elk van de bijwerkingen lastig voor u was *

Beantwoord deze vraag alleen als aan de volgende voorwaarden is voldaan:
Antwoord was 'Ja' bij vraag ' [HC2]' (Werd u de afgelopen 5 jaar behandeld voor uw psychotische problematiek?) *en* Antwoord ONGELIJK 'Geen nadelige effecten' bij vraag '

[HC4]' (Welke bijwerking(en) heeft u tijdens of na de behandeling voor uw psychotische problematiek gehad in de afgelopen 5 jaar?)
Kies het toepasselijke antwoord voor elk onderdeel:

	Helemaal niet lastig	Licht lastig	Matig lastig	Erg lastig	Extreem lastig	Ik weet het niet
**Gewichtstoename**						
**Hyperglycemie (te veel suiker in het bloed) of diabetes**						
**Hoge cholesterol (overtollige vetten in het bloed)**						
**Tekort aan witte bloedcellen**						
**Rusteloosheid, moeite met stilzitten, behoefte om te bewegen**						
**Onvrijwillige en soms pijnlijke spiersamentrekking**						
**Beven en/of gezichtsstijfheid**						
**Onwillekeurige gezichtsbewegingen, zoals kauwen, tong uitsteken of knipperen**						
**Vermoeidheid of slaperigheid**						
**Duizeligheid**						
**Droge mond of ogen**						
**Misselijkheid**						
**Constipatie**						
**Myocarditis (ontsteking van de hartspier)**						
**Seksuele dysfunctie (bv. verminderd libido)**						
**Geheugenproblemen of "mentale mist"**						
**Emotionele afvlakking (gebrek aan motivatie, gebrek aan interesse)**						
**Geen nadelige effecten**						

Onder lastig verstaan we: onaangenaam, vermoeiend of beperkend, iets wat hinder veroorzaakt of een fysiek of mentaal ongemak

Hoe lastig is de behandeling die u de afgelopen 5 jaar voor uw psychotische problematiek kreeg voor u geweest, <u>afgezien van de</u>

bijwerkingen? Bijvoorbeeld vanwege de toediening via injectie, de noodzaak om naar het ziekenhuis te gaan voor de behandeling, een strikt medicatieschema, de noodzaak om de medicatie in de koelkast te bewaren, etc.? *

Beantwoord deze vraag alleen als aan de volgende voorwaarden is voldaan:
((HC2.NAOK == "A1"))
Kies één van de volgende antwoorden
Kies één van de volgende mogelijkheden:

- Helemaal niet lastig
- Licht lastig
- Matig lastig
- Erg lastig
- Extreem lastig
- Ik weet het niet

Sympto(o)m(en) gerelateerd aan uw psychotische problematiek

Sinds u opgevolgd of behandeld wordt voor de psychotische problematiek, welke van de volgende symptomen hebt u ervaren en hoe lastig waren ze voor u? Houd geen rekening met de ongewenste effecten van de behandeling. *

Kies het toepasselijke antwoord voor elk onderdeel:

	Ik heb dit symptoom niet gehad	Helemaal niet lastig	Licht lastig	Matig lastig	Erg lastig	Extreem lastig	Ik weet het niet
De indruk dat iemand speciaal voor jou iets op de televisie zegt of in de krant schrijft, de indruk dat iemand je opzettelijk kwaad wil doen, de indruk dat er een complot tegen je is, of de indruk dat je gedachten als door een echo worden herhaald.							
Iets horen, zien of voelen dat er niet is. Bijvoorbeeld een of meer stemmen horen terwijl je helemaal alleen bent; objecten, mensen of dieren zien die andere mensen niet kunnen zien.							

	Ik heb dit symptoom niet gehad	Helemaal niet lastig	Licht lastig	Matig lastig	Erg lastig	Extreem lastig	Ik weet het niet
Een gevoel van invloed, controle of passiviteit. Bijvoorbeeld de indruk dat je gedachten in je hoofd hebt die je niet als de jouwe herkent. De indruk onder controle te staan van een kracht of macht buiten jezelf.							
Desorganisatie van het denken, zoals het gevoel dat je geest leeg is, of dat je gedachten uit je hoofd zijn gehaald, of dat je gedachten in je hoofd hebt die je niet als de jouwe herkent.							
Desorganisatie van het gedrag. Bv. het gevoel dat je je uiterlijk of je persoonlijke hygiëne verwaarloost, of het gevoel dat je nooit afmaakt waar je mee bezig bent.							
Onvermogen om interesse of plezier te voelen, gebrek aan motivatie of enthousiasme, het gevoel dat je emoties afgestompt zijn.							
Sociaal isolement, of het gevoel dat je geen interesse hebt om met andere mensen om te gaan.							
Agitatie of gevoelens van spanning							
Concentratieproblemen							
Vermoeidheid of uitputting							
Angst							
Gevoelens van neerslachtigheid, depressie of verdriet							
Stress							
Gedrag of verlangen om zichzelf pijn te doen of te verwonden							

	Ik heb dit symptoom niet gehad	Helemaal niet lastig	Licht lastig	Matig lastig	Erg lastig	Extreem lastig	Ik weet het niet
Het gevoel niet meer te willen leven							
Stigmatisatie of gevoelens van discriminatie							
Verlies van zelfvertrouwen							
Andere							

In welke mate heeft uw psychotische problematiek uw seksleven verstoord? *

Kies één van de volgende antwoorden
Kies één van de volgende mogelijkheden:

- Helemaal niet
- Licht
- Matig
- Zwaar
- Extreem
- Ik weet het niet
- Niet van toepassing
- Ik wil niet antwoorden

In welke mate heeft uw psychotische problematiek een invloed gehad op uw vruchtbaarheid en voortplanting (d.w.z. op uw vermogen of beslissing om op natuurlijke wijze kinderen te verwekken of een zwangerschap te voltooien)?

Bijvoorbeeld: IVF (In Vitro Fertilisatie), genetische screening, sperma-/eiceldonatie, uitstel van zwangerschap of ervoor kiezen geen biologische kinderen te krijgen vanwege uw psychotische problematiek?

*

Kies één van de volgende antwoorden
Kies één van de volgende mogelijkheden:

- Helemaal niet
- Licht
- Matig
- Zwaar
- Extreem
- Ik weet het niet

- Niet van toepassing (bijv. Nog niet overwogen om kinderen te krijgen, of niet getroffen door de psychotische problematiek toen ik op de leeftijd was om kinderen te krijgen)
- Ik wil niet antwoorden

Medische behandeling van de psychotische problematiek

Met 'medische behandeling' of zorg bedoelen we alle afspraken, behandelingen en ondersteuning die door zorgverleners worden aangeboden om uw gezondheidstoestand op vlak van uw psychotische problematiek te beheren en te verbeteren.

Heeft u het gevoel dat uw zorg in de loop van de tijd goed georganiseerd en gepland is? *

Kies één van de volgende antwoorden
Kies één van de volgende mogelijkheden:

- Altijd
- Vaak
- Soms
- Zelden
- Nooit
- Ik weet het niet

Hebt u één of meer zorgverleners die verantwoordelijk zijn voor de coördinatie van de verschillende medische diensten waarvan u gebruik maakt? *

Kies één van de volgende antwoorden
Kies één van de volgende mogelijkheden:

- Ja
- Neen
- Ik weet het niet
- Niet van toepassing: de opvolging gebeurt door één dienst, zonder behoefte aan coördinatie

Bijvoorbeeld een referentie arts of verpleegkundige die u opvolgt of doorverwijst, en die de communicatie vergemakkelijkt tussen de verschillende specialisten of diensten die u raadpleegt.

In welke mate bezoekt u dezelfde zorgverlener(s) tijdens uw regelmatige afspraken of controles? *

Kies één van de volgende antwoorden

Kies één van de volgende mogelijkheden:

- Altijd
- Vaak
- Soms
- Zelden
- Nooit
- Ik weet het niet

In welke mate krijgt u voldoende <u>nuttige en begrijpelijke</u> informatie van uw zorgverleners?

*

Kies één van de volgende antwoorden
Kies één van de volgende mogelijkheden:

- Altijd
- Vaak
- Soms
- Zelden
- Nooit
- Ik weet het niet

Met 'nuttig' bedoelen we actuele en coherente informatie die u helpt uw psychotische problematiek beter te begrijpen en aan te pakken.

Wordt u, zoveel als u wenst, betrokken bij de beslissingen over uw zorg of behandeling? *

Kies één van de volgende antwoorden
Kies één van de volgende mogelijkheden:

- Ja, helemaal
- Ja, tot op zekere hoogte
- Nee, niet echt
- Nee, helemaal niet
- Nee, maar ik wilde er toch niet bij betrokken worden
- Niet van toepassing: er worden geen beslissingen genomen over behandeling
- Ik weet het niet

Toegang tot gezondheidszorg

Had u <u>in de afgelopen 12 maanden</u> bepaalde zorg nodig voor uw psychotische problematiek die u niet heeft gekregen?
Dit kan een consultatie, behandeling, medische test, revalidatie of iets anders zijn. *

Kies één van de volgende antwoorden
Kies één van de volgende mogelijkheden:

- Ja
- Neen
- Ik weet het niet

Om welke reden(en) heeft u zorg die u nodig had niet gekregen?

*

Beantwoord deze vraag alleen als aan de volgende voorwaarden is voldaan:
Antwoord was 'Ja' bij vraag ' [HC12]' (Had u in de afgelopen 12 maanden bepaalde zorg nodig voor uw psychotische problematiek die u niet heeft gekregen?Dit kan een consultatie, behandeling, medische test, revalidatie of iets anders zijn.)
Alles selecteren wat van toepassing is
Kies alle voor u geldende mogelijkheden:

- Afstand tussen mijn woonst en de plaats van zorg
- Vervoersproblemen (bijv.: geen openbaar vervoer in de buurt, geen eigen voertuig, niet in staat om eigen voertuig te besturen, niemand om u naar de plaats van zorg te brengen)
- Gebrek aan bekwaam personeel om u de zorg te bieden die u nodig had
- Lange wachttijden
- Moeilijkheden om de zorg te betalen
- Angst voor medische-, ziekenhuis-, of andere onderzoeken
- Tijdsgebrek door werk, kinderen of andere
- Gebrek aan informatie
- Taalproblemen
- Overige:

Impact van de psychotische problematiek op onderwijs en werk

In welke mate werd uw school- of studietijd beïnvloed door uw psychotische problematiek, in termen van aanwezigheid, prestaties, succes of studiekeuze? *

Kies één van de volgende antwoorden
Kies één van de volgende mogelijkheden:

- Helemaal niet
- Licht
- Matig
- Zwaar
- Extreem
- Ik weet het niet

- Niet van toepassing: de psychotische problematiek deed zich voor nadat school/studie was afgerond

Hebt u door uw psychotische problematiek uw werktijd verminderd? *

Beantwoord deze vraag alleen als aan de volgende voorwaarden is voldaan:
((D10.NAOK == "A1"))
Kies één van de volgende antwoorden
Kies één van de volgende mogelijkheden:

- Ja, ik werk minder dan vroeger door mijn psychotische problematiek
- Ja, ik ben gestopt met werken vanwege mijn psychotische problematiek
- Nee, de psychotische problematiek heeft geen invloed op mijn werkuren
- Ik weet het niet
- Overige

Hoe lang kon u niet werken vanwege uw psychotische problematiek? *

Kies één van de volgende antwoorden
Kies één van de volgende mogelijkheden:

- Voor minder dan 1 maand
- Voor 1 tot 3 maanden
- Voor 4 maanden tot 1 jaar
- Voor meer dan 1 jaar
- Ik weet het niet

Geef een ruwe schatting van het TOTAAL van alle ziekteverzuimen, al dan niet aaneengesloten.

Bijvoorbeeld: als u eerst 1 maand en daarna 3 maanden niet heeft gewerkt, vink dan « Voor 4 maanden tot 1 jaar » aan.

Geef bij benadering het aantal jaren aan: *

Beantwoord deze vraag alleen als aan de volgende voorwaarden is voldaan:
Antwoord was 'Voor meer dan 1 jaar ' bij vraag ' [S3]' (Hoe lang kon u niet werken vanwege uw psychotische problematiek?)
In dit veld mogen alleen cijfers ingevoerd worden.
Vul uw antwoord hier in:

-

De volgende vraag gaat over de afgelopen zeven dagen, vandaag niet meegerekend. *

Beantwoord deze vraag alleen als aan de volgende voorwaarden is voldaan:

Antwoord was 'Nee, de psychotische problematiek heeft geen invloed op mijn werkuren' *of* 'Ja, ik werk minder dan vroeger door mijn psychotische problematiek' bij vraag ' [S2]' (Hebt u door uw psychotische problematiek uw werktijd verminderd?)

In welke mate heeft uw psychotische problematiek de afgelopen zeven dagen uw productiviteit op het werk beïnvloed? Denk aan de dagen waarop u beperkt werd in de hoeveelheid of het soort werk dat u kon doen, de dagen waarop u minder bereikte dan u had gewild, of de dagen waarop u uw werk niet zo zorgvuldig kon doen als gewoonlijk. Indien de psychotische problematiek weinig invloed had op uw werk, kies dan een laag cijfer. Kies een hoog cijfer als de ziekte een grote invloed had op uw werk. *

Beantwoord deze vraag alleen als aan de volgende voorwaarden is voldaan:
Antwoord was 'Nee, de psychotische problematiek heeft geen invloed op mijn werkuren' *of* 'Ja, ik werk minder dan vroeger door mijn psychotische problematiek' bij vraag ' [S2]' (Hebt u door uw psychotische problematiek uw werktijd verminderd?)
Elk antwoord moet tussen 0 en 10 zijn
In deze velden mogen alleen gehele getallen worden ingevoerd.
Vul uw antwoord(en) hier in:

- |

Denk alleen aan de mate waarin de psychotische problematiek uw productiviteit heeft beïnvloed terwijl u werkt. Klik op de cursor om deze tot op de gewenste waarde te schuiven

Financieel gevolg

In welke mate heeft uw psychotische problematiek of medische behandeling u financiële problemen bezorgd? *

Kies één van de volgende antwoorden
Kies één van de volgende mogelijkheden:

- Helemaal niet
- Licht
- Matig
- Zwaar
- Extreem
- Ik weet het niet

Impact op sociale relaties en ondersteuningsbehoeften

In welke mate belemmert uw psychotische problematiek uw relaties met uw familie, partner, vrienden of buren? *

Kies één van de volgende antwoorden
Kies één van de volgende mogelijkheden:

- Helemaal niet
- Licht
- Matig
- Zwaar
- Extreem
- Ik weet het niet

Welke vormen van extra ondersteuning heeft u nodig omwille van uw psychotische problematiek, maar heeft u tot nu toe nog niet kunnen krijgen?

*

Alles selecteren wat van toepassing is
Kies alle voor u geldende mogelijkheden:

- Geen extra ondersteuning
- Logistieke ondersteuning (bv. schoonmaken, koken, boodschappen doen, vervoer, kinderopvang)
- Praten met andere patiënten met een psychotische problematiek
- Psychologische ondersteuning
- Medische ondersteuning
- Administratieve of sociale bijstand
- Ondersteuning bij aanpassen van de omgeving of aangepast materiaal
- Ondersteuning bij terugkeer naar werk
- Bijles en studiebegeleiding
- Spirituele of religieuze begeleiding
- Ik weet het niet
- Overige:

Prioritaire behoeften

Welke aspecten van uw psychotische problematiek of de behandeling ervan moeten volgens u prioritair verbeterd worden? Rangschik elk aspect volgens het belang dat u eraan hecht. *

Kies het toepasselijke antwoord voor elk onderdeel:

	Helemaal geen prioriteit	Lage prioriteit	Matige prioriteit	Hoge prioriteit	Zeer hoge prioriteit	Ik weet het niet
De impact van uw psychotische problematiek op uw algemene levenskwaliteit (impact op mobiliteit,						

	Helemaal geen prioriteit	Lage prioriteit	Matige prioriteit	Hoge prioriteit	Zeer hoge prioriteit	Ik weet het niet
**autonomie, uzelf kunnen aankleden en wassen, ongemak of pijn, angst of depressie; jaren geleefd met een handicap)**						
**De impact op uw fysieke gezondheid (fysieke symptomen, ongemak of pijn gerelateerd aan de psychotische problematiek)**						
**De impact op uw psychologische gezondheid (psychologische symptomen, angst of depressie gerelateerd aan de psychotische problematiek)**						
**De invloed op uw onafhankelijkheid (invloed op uw mobiliteit, uw vermogen om u te wassen en aan te kleden; vermogen om dagelijkse activiteiten uit te voeren zoals werk, studie, huishouden, gezinsactiviteiten of vrije tijd)**						
**De invloed op uw seksuele en reproductieve leven**						
**De invloed op uw levensverwachting**						
**De invloed op uw scholing of studie**						
**De invloed op uw werk**						
**De financiële impact**						
**De impact op uw sociale leven (relaties met uw familie, partner of vrienden; behoefte aan extra ondersteuning - administratief, sociaal, ezv.).**						
**De kwaliteit van uw zorg (snelheid van diagnose, coördinatie en continuïteit van zorg, informatie**						

	Helemaal geen prioriteit	Lage prioriteit	Matige prioriteit	Hoge prioriteit	Zeer hoge prioriteit	Ik weet het niet
ontvangen door zorgverleners, betrokkenheid bij beslissingen over uw zorg of behandeling)						
Toegang tot gezondheidszorg (zorg krijgen wanneer u die nodig hebt, beschikbaarheid van behandeling)						
Wetenschappelijke kennis (bijv. op het gebied van diagnose, behandeling of prognose)						
Effectiviteit van de behandeling (het vermogen van de behandeling om de symptomen of de kwaliteit van leven te verbeteren)						
Moeilijkheid van de behandeling (op het gebied van bijwerkingen of logistiek/administratie van de behandeling)						

Andere belangrijke behoeften

Wilt u ons vertellen over één of meer andere belangrijke behoeften die verband houden met uw psychotische problematiek die niet aan bod kwamen in de vragenlijst? *

Kies één van de volgende mogelijkheden:

- Ja
- Nee

Welke behoeften werden niet behandeld in de vragenlijst?

Beantwoord deze vraag alleen als aan de volgende voorwaarden is voldaan:
Antwoord was 'Ja' bij vraag ' [G1]' (Wilt u ons vertellen over één of meer andere belangrijke behoeften die verband houden met uw psychotische problematiek die niet aan bod kwamen in de vragenlijst?)
Vul uw antwoord hier in:

Impact op verzorgers

Als iemand die de vragenlijst invult voor iemand met een psychotische problematiek: beschouwt u zichzelf als een 'mantelzorger'?

Met andere woorden, u helpt deze persoon omwille van zijn/haar psychotische problematiek, op regelmatige basis en in een niet-professionele hoedanigheid. Bijvoorbeeld een ouder die een kind helpt, een gezinslid of een vriend die een naaste helpt.

*****

Kies één van de volgende mogelijkheden:

- Ja
- Nee

In relatie tot de persoon die u helpt, bent u: *

Beantwoord deze vraag alleen als aan de volgende voorwaarden is voldaan:
Antwoord was 'Ja' bij vraag ' [CG1]' (Als iemand die de vragenlijst invult voor iemand met een psychotische problematiek: beschouwt u zichzelf als een 'mantelzorger'? Met andere woorden, u helpt deze persoon omwille van zijn/haar psychotische problematiek, op regelmatige basis en in een niet-professionele hoedanigheid. Bijvoorbeeld een ouder die een kind helpt, een gezinslid of een vriend die een naaste helpt.)
Kies één van de volgende antwoorden
Kies één van de volgende mogelijkheden:

- Een familielid (bijvoorbeeld: ouder, kind, grootouder, ...)
- Een nauwe relatie (bijvoorbeeld: vriend, buur, ...)
- Overige

Met welk geslacht identificeert u zich?

Beantwoord deze vraag alleen als aan de volgende voorwaarden is voldaan:
Antwoord was 'Ja' bij vraag ' [CG1]' (Als iemand die de vragenlijst invult voor iemand met een psychotische problematiek: beschouwt u zichzelf als een 'mantelzorger'? Met andere woorden, u helpt deze persoon omwille van zijn/haar psychotische problematiek, op regelmatige basis en in een niet-professionele hoedanigheid. Bijvoorbeeld een ouder die een kind helpt, een gezinslid of een vriend die een naaste helpt.)
Kies één van de volgende antwoorden
Kies één van de volgende mogelijkheden:

- Man
- Vrouw
- Andere

In welk jaar bent u geboren? *

Beantwoord deze vraag alleen als aan de volgende voorwaarden is voldaan:
Antwoord was 'Ja' bij vraag ' [CG1]' (Als iemand die de vragenlijst invult voor iemand met een psychotische problematiek: beschouwt u zichzelf als een 'mantelzorger'? Met andere woorden, u helpt deze persoon omwille van zijn/haar psychotische problematiek, op regelmatige basis en in een niet-professionele hoedanigheid. Bijvoorbeeld een ouder die een kind helpt, een gezinslid of een vriend die een naaste helpt.)
In dit veld mogen alleen cijfers ingevoerd worden.
Uw antwoord mag minimaal 1920 zijn
Vul uw antwoord hier in:

-

Hoeveel tijd hebt u tijdens de afgelopen week besteed aan het helpen van de persoon voor activiteiten die hij/zij normaal gesproken zelf zou hebben gedaan als hij/zij in goede gezondheid was?

Bijvoorbeeld voor persoonlijke verzorging (bijvoorbeeld aan- en uitkleden, naar het toilet gaan, ...), huishoudelijke taken (bijvoorbeeld eten klaarmaken, schoonmaken, ...) of praktische ondersteuning (bijvoorbeeld begeleiden naar medische of andere afspraken, ...)

Beantwoord deze vraag alleen als aan de volgende voorwaarden is voldaan:
Antwoord was 'Ja' bij vraag ' [CG1]' (Als iemand die de vragenlijst invult voor iemand met een psychotische problematiek: beschouwt u zichzelf als een 'mantelzorger'? Met andere woorden, u helpt deze persoon omwille van zijn/haar psychotische problematiek, op regelmatige basis en in een niet-professionele hoedanigheid. Bijvoorbeeld een ouder die een kind helpt, een gezinslid of een vriend die een naaste helpt.)
In dit veld mogen alleen cijfers ingevoerd worden.
Vul uw antwoord hier in:

- uren in de afgelopen week.

In welke mate wordt volgens u uw fysieke en/of psychologische gezondheid beïnvloed door uw rol als mantelzorger? *

Beantwoord deze vraag alleen als aan de volgende voorwaarden is voldaan:
Antwoord was 'Ja' bij vraag ' [CG1]' (Als iemand die de vragenlijst invult voor iemand met een psychotische problematiek: beschouwt u zichzelf als een 'mantelzorger'? Met andere woorden, u helpt deze persoon omwille van zijn/haar psychotische problematiek, op regelmatige basis en in een niet-professionele hoedanigheid. Bijvoorbeeld een ouder die een kind helpt, een gezinslid of een vriend die een naaste helpt.)
Kies één van de volgende antwoorden
Kies één van de volgende mogelijkheden:

- Helemaal niet

- Licht
- Matig
- Zwaar
- Extreem
- Ik weet het niet

In welke mate wordt volgens u uw sociale leven beïnvloed door uw rol als mantelzorger? *

Beantwoord deze vraag alleen als aan de volgende voorwaarden is voldaan:
Antwoord was 'Ja' bij vraag ' [CG1]' (Als iemand die de vragenlijst invult voor iemand met een psychotische problematiek: beschouwt u zichzelf als een 'mantelzorger'? Met andere woorden, u helpt deze persoon omwille van zijn/haar psychotische problematiek, op regelmatige basis en in een niet-professionele hoedanigheid. Bijvoorbeeld een ouder die een kind helpt, een gezinslid of een vriend die een naaste helpt.)
Kies één van de volgende antwoorden
Kies één van de volgende mogelijkheden:

- Helemaal niet
- Licht
- Matig
- Zwaar
- Extreem
- Ik weet het niet

Vervolg van het onderzoek

We willen graag meer weten over de behoeften en moeilijkheden die u ervaart of hebt ervaren in de context van uw psychotische problematiek. Als u dat wilt, kunt u deelnemen aan een één-op-één interview (online of face-to-face met een onderzoeker) om uw ervaringen en behoeften in meer detail te bespreken. Dit interview vindt vertrouwelijk plaats en heeft geen invloed op de zorg die u ontvangt van zorgverleners. Er zullen naar verwachting 24 patiënten worden geïnterviewd. Dit individuele interview zal ongeveer anderhalf uur duren.

Wilt u deelnemen aan een persoonlijk interview?

*****

Kies één van de volgende mogelijkheden:

- Ja
- Nee

Als u geïnteresseerd bent in een persoonlijk gesprek, geef ons dan uw contactgegevens (telefoonnummer en e-mailadres) zodat we met u contact kunnen opnemen. *

Beantwoord deze vraag alleen als aan de volgende voorwaarden is voldaan:
Antwoord was 'Ja' bij vraag ' [Follow]' (We willen graag meer weten over de behoeften en moeilijkheden die u ervaart of hebt ervaren in de context van uw psychotische problematiek. Als u dat wilt, kunt u deelnemen aan een één-op-één interview (online of face-to-face met een onderzoeker) om uw ervaringen en behoeften in meer detail te bespreken. Dit interview vindt vertrouwelijk plaats en heeft geen invloed op de zorg die u ontvangt van zorgverleners. Er zullen naar verwachting 24 patiënten worden geïnterviewd. Dit individuele interview zal ongeveer anderhalf uur duren. Wilt u deelnemen aan een persoonlijk interview?)
Vul uw antwoord(en) hier in:

- e-mail adres
- Bevestiging e-mail adres
- GSM nummer

Hartelijk dank voor uw deelname.

Iemand van onze onderzoekers zal u binnen een paar weken informeren over een eventueel persoonlijk gesprek met een onderzoeker.

Beantwoord deze vraag alleen als aan de volgende voorwaarden is voldaan:
Antwoord was 'Ja' bij vraag ' [Follow]' (We willen graag meer weten over de behoeften en moeilijkheden die u ervaart of hebt ervaren in de context van uw psychotische problematiek. Als u dat wilt, kunt u deelnemen aan een één-op-één interview (online of face-to-face met een onderzoeker) om uw ervaringen en behoeften in meer detail te bespreken. Dit interview vindt vertrouwelijk plaats en heeft geen invloed op de zorg die u ontvangt van zorgverleners. Er zullen naar verwachting 24 patiënten worden geïnterviewd. Dit individuele interview zal ongeveer anderhalf uur duren. Wilt u deelnemen aan een persoonlijk interview?)

Bedankt voor uw interesse in deze studie.

De resultaten van deze studie zullen begin 2026 gepubliceerd worden op de site van het KCE (www.kce.fgov.be) en op de site van NEED (www.health-needs.eu).

De gegevens van de personen die aan de enquête deelnemen, worden zodanig gegroepeerd dat het onmogelijk is om een individu te identificeren op basis van de gepubliceerde gegevens. Uw privacy en de vertrouwelijkheid van uw antwoorden zijn dus volledig beschermd. Indien het invullen van deze vragenlijst of het nadenken over uw psychotische problematiek ongemak of emotioneel leed heeft veroorzaakt, raden wij u aan om hulp te zoeken. U kan terecht bij één van onze onderzoekers, Margaux Sageot, op het nummer 04 73 73 36 06. U kunt hiervoor ook terecht bij uw huisarts, psychiater of psycholoog.

In geval van dringende nood kunt u 112 bellen. Indien u nood heeft aan een anoniem gesprek kunt u 106 bellen.

Verzend uw enquête.

Bedankt voor uw deelname aan deze enquête.

Questionnaire NEED

ENQUÊTE SUR LES BESOINS NON-RENCONTRÉS DES PATIENTS ADULTES ATTEINTS D'UN TROUBLE PSYCHOTIQUE

Merci d'avoir accepté de participer à notre enquête.

Notre étude vise à identifier les besoins des patients atteints d'un trouble psychotique afin d'améliorer leur accompagnement.

Ce questionnaire vous concerne si:

- Vous êtes atteint d'un trouble psychotique (par exemple schizophrénie, trouble schizo-affectif, ou autre trouble psychotique)
- Vous avez 18 ans ou plus
- Vous habitez en Belgique (résidence principale)

Ou

- Vous vous exprimez au nom d'une personne adulte atteinte d'un trouble psychotique diagnostiquée dont vous êtes le représentant légal. Cette personne habite en Belgique, et n'est pas en mesure de répondre elle-même.

L'étude vise à mesurer les besoins non-rencontrés des personnes atteintes d'un trouble psychotique. Cela peut aider à améliorer la prise en charge des patients ayant un trouble psychotique. Certaines questions peuvent sembler moins adaptées à votre situation personnelle ou paraître non pertinentes. Néanmoins, elles sont importantes pour pouvoir comparer les besoins de personnes atteintes d'autres maladies. Nous vous prions donc de répondre aux questions aussi précisément que possible afin de bien cerner vos besoins.

Les résultats de cette étude seront publiés sur le site Internet du KCE (www.kce.fgov.be) et sur le site de NEED (www.health-needs.eu) début 2026.

Avant d'accepter de participer à cette enquête, nous vous demandons de lire les informations suivantes sur la confidentialité de vos données, et de donner votre consentement à l'utilisation de vos données.

Il y a 77 questions dans ce questionnaire.

Informations générales

Vous remplissez ce questionnaire *

Veuillez sélectionner une réponse ci-dessous.
Veuillez sélectionner une seule des propositions suivantes :

- Pour vous-même

- Pour une autre personne dont vous êtes le représentant légal. (La personne que vous représentez n'est pas capable de répondre au questionnaire pour le moment. En cochant cette case, vous participez à cette étude en fonction de sa volonté probable de participer.)

A partir de maintenant veuillez répondre à toutes les questions comme si vous étiez la personne atteinte du trouble psychotique. Indiquez "je ne sais pas" si vous ne savez pas comment la personne répondrait.

Répondre à cette question seulement si les conditions suivantes sont réunies :
La réponse était 'Pour une autre personne dont vous êtes le représentant légal. (La personne que vous représentez n'est pas capable de répondre au questionnaire pour le moment. En cochant cette case, vous participez à cette étude en fonction de sa volonté probable de participer.)' à la question ' [D1]' (Vous remplissez ce questionnaire)

À quel genre vous identifiez-vous ? *

Veuillez sélectionner une réponse ci-dessous.
Veuillez sélectionner une seule des propositions suivantes :

- Homme
- Femme
- Autre

Quelle est votre année de naissance ? *

Votre réponse doit être d'au moins 1920
Seul un nombre entier peut être inscrit dans ce champ.
Veuillez écrire votre réponse ici :

-

Merci pour votre participation.

Malheureusement, vous ne rentrez pas dans les conditions pour pouvoir poursuivre cette étude.

Répondre à cette question seulement si les conditions suivantes sont réunies :
((D3.NAOK > 2007))

Dans quelle province habitez-vous ? *

Répondre à cette question seulement si les conditions suivantes sont réunies :
(((! is_empty(D3.NAOK) && (D3.NAOK <= 2007))))
Veuillez sélectionner une réponse ci-dessous.
Veuillez sélectionner une seule des propositions suivantes :

- Anvers

- Brabant wallon
- Bruxelles
- Hainaut
- Liège
- Limbourg
- Luxembourg
- Namur
- Flandre orientale
- Brabant flamand
- Flandre occidentale
- Je n’habite pas en Belgique
- Je ne sais pas

Merci pour votre participation.

Malheureusement, vous ne rentrez pas dans les conditions pour pouvoir poursuivre cette étude.

Répondre à cette question seulement si les conditions suivantes sont réunies :
(((! is_empty(D3.NAOK) && (D3.NAOK <= 2007))) and (D4.NAOK == "AO12"))

Informations générales

Quel est le diplôme ou le niveau d’étude le plus élevé que vous avez obtenu jusqu’à présent? *

Veuillez sélectionner une réponse ci-dessous.
Veuillez sélectionner une seule des propositions suivantes :

- Pas de diplôme ou enseignement primaire
- Enseignement secondaire inférieur
- Enseignement secondaire supérieur
- Enseignement supérieur de type court (p.ex. Bachelier)
- Enseignement supérieur de type long (p.ex. Master)
- Doctorat
- Je ne sais pas
- Autre

Vous vivez ... *

Veuillez sélectionner une réponse ci-dessous.
Veuillez sélectionner une seule des propositions suivantes :

- Seul(e)
- Seul(e) avec enfant(s)
- En couple (sans enfants)
- En couple (avec enfants)
- Avec un ou deux parents

- En colocation ou habitat partagé
- En maison de soins psychiatriques
- En habitation protégée
- En logement supervisé
- A l'hôpital (temporairement)
- En maison de repos
- Je ne sais pas
- Autre

Quel est votre pays de naissance ? *

Veuillez sélectionner une réponse ci-dessous.
Veuillez sélectionner une seule des propositions suivantes :

- Belgique
- Autre pays
- Je ne sais pas

Sélectionnez le pays concerné *

Répondre à cette question seulement si les conditions suivantes sont réunies :
La réponse était 'Autre pays' à la question ' [D7]' (Quel est votre pays de naissance ?)
Veuillez sélectionner une réponse ci-dessous.
Veuillez sélectionner une seule des propositions suivantes :

- Afghanistan
- Afrique du Sud
- Albanie
- Algérie
- Allemagne
- Andorre
- Angola
- Anguilla
- Antarctique
- Antigua et Barbuda
- Arabie Saoudite
- Argentine
- Arménie
- Aruba
- Australie
- Autriche
- Azerbaïdjan
- Bahamas
- Bahreïn
- Bangladesh
- Barbade
- Bélize
- Bénin
- Bermudes

- Bhoutan
- Biélorussie
- Bolivie
- Bonaire
- Bosnie - Herzégovine
- Botswana
- Brésil
- Brunei
- Bulgarie
- Burkina Faso
- Burundi
- Cambodge
- Cameroun
- Canada
- Cap-Vert
- Chili
- Chine
- Chypre
- Colombie
- Comores
- Congo (-Brazzaville)
- Congo (République Démocratique)
- Corée du Nord
- Corée du Sud (République de Corée)
- Costa Rica
- Côte d'Ivoire
- Croatie
- Cuba
- Curaçao
- Danemark et îles Féroé
- Djibouti
- Dominique
- Egypte
- El Salvador
- Emirats Arabes Unis
- Equateur
- Erythrée
- Espagne et Iles Canaries
- Estonie
- eSwatini (Swaziland)
- États-Unis d'Amérique
- Ethiopie
- Fidji
- Finlande
- France
- Gabon
- Gambie
- Géorgie
- Ghana

- Gibraltar
- Grèce
- Grenade
- Groenland
- Guadeloupe
- Guam
- Guatemala
- Guinée-Bissau
- Guinée (Conakry)
- Guinée equatoriale
- Guyana (Georgetown)
- Guyane (Française)
- Haïti
- Honduras
- Hong Kong
- Hongrie
- Ile Christmas
- Iles Caïmans
- Iles Cook
- Iles Malouines
- Iles Marshall
- Iles Salomon
- Iles Vierges (américaines)
- Iles Vierges (britanniques)
- Inde
- Indonésie
- Irak
- Iran
- Irlande
- Islande
- Israël
- Italie
- Jamaïque
- Japon
- Jordanie
- Kazakhstan
- Kenya
- Kirghizistan
- Kiribati
- Kosovo
- Koweït
- Laos
- Lesotho
- Lettonie
- Liban
- Libéria
- Libye
- Liechtenstein
- Lituanie

- Luxembourg
- Macao (Chine)
- Macédoine du Nord
- Madagascar
- Malaisie
- Malawi
- Maldives
- Mali
- Malte
- Mariannes du Nord (Iles, américaines)
- Maroc
- Martinique
- Maurice
- Mauritanie
- Mayotte
- Mexique
- Micronesie
- Moldova
- Monaco
- Mongolie
- Monténégro
- Montserrat (britannique)
- Mozambique
- Myanmar
- Namibie
- Nauru
- Népal
- Nicaragua
- Niger
- Nigéria
- Niue
- Norvège
- Nouvelle-Calédonie
- Nouvelle-Zélande
- Oman
- Ouganda
- Ouzbékistan
- Pakistan
- Palau
- Palestine
- Panama
- Papouasie-Nouvelle-Guinée
- Paraguay
- Pays-Bas
- Pérou
- Philippines
- Pitcairn
- Pologne
- Polynésie (français)

- Porto Rico
- Portugal, Açores, Madère
- Qatar
- République Centrafricaine
- République Dominicaine
- République tchèque
- Réunion
- Roumanie
- Royaume-Uni
- Russie
- Rwanda
- Sahara occidental
- Saint-Barthélemy
- Saint-Christophe-et-Niévès
- Saint-Eustache et Saba
- Saint-Marin
- Saint-Martin (France)
- Saint-Martin (Pays-Bas)
- Saint-Pierre-et-Miquelon
- Saint-Vincent-et-les-Grenadines
- Sainte-Hélène - Tristan Da Cunha - Ascension
- Sainte-Lucie
- Samoa
- Samoa (Américain)
- Sao Tomé et Principe
- Sénégal
- Serbie
- Seychelles
- Sierra Leone
- Singapour
- Slovaquie
- Slovénie
- Somalie
- Soudan
- Soudan du Sud
- Sri Lanka
- Suède
- Suisse
- Suriname
- Syrie
- Tadjikistan
- Taiwan
- Tanzanie
- Tchad
- Thaïlande
- Timor oriental
- Togo
- Tokelau (Nouvelle-Zélande)
- Tonga

- Trinité et Tobago
- Tunisie
- Turkménistan
- Turques-et-Caïques (britanniques)
- Turquie
- Tuvalu
- Ukraine
- Uruguay
- Vanuatu
- Venezuela
- Vietnam
- Wallis-et-Futuna
- Yémen
- Zambie
- Zimbabwe
- Je ne sais pas
- Autre

Quel était votre <u>principal</u> statut professionnel <u>juste avant le début de votre trouble psychotique</u> ? *

Veuillez sélectionner une réponse ci-dessous.
Veuillez sélectionner une seule des propositions suivantes :

- Emploi rémunéré (employé ou indépendant)
- Demandeur/demandeuse d'emploi, chômage, CPAS
- Homme/femme au foyer, sans revenus
- Etudiant(e)
- Pensionné(e)
- Invalidité
- Je ne sais pas
- Autre

Depuis le début de vos premiers symptômes psychotiques, avez-vous eu un emploi rémunéré?

*

Répondre à cette question seulement si les conditions suivantes sont réunies :
La réponse n'était PAS 'Pensionné(e)' à la question ' [D9]' (Quel était votre principal statut professionnel juste avant le début de votre trouble psychotique ?)
Veuillez sélectionner une réponse ci-dessous.
Veuillez sélectionner une seule des propositions suivantes :

- Oui
- Non
- Je ne sais pas

Considérez comme "premiers symptômes psychotiques" les premières manifestations de votre trouble psychotique, telles qu'entendre des voix ou des bruits qui n'existent pas selon les autres ou de voir des choses que les autres ne voient pas, avoir des pensées qui ne correspondent pas à la réalité perçue par les autres, ou avoir l'idée que quelqu'un cherche volontairement à vous nuire. Si vous ne savez pas quand vos symptômes ont commencé, veuillez à la place considérer le moment de votre premier contact avec les soins psychiatriques.

Actuellement, quel est votre principal statut professionnel? *

Veuillez sélectionner une réponse ci-dessous.
Veuillez sélectionner une seule des propositions suivantes :

- Emploi rémunéré (employé ou indépendant)
- Demandeur/demandeuse d'emploi, chômage, CPAS
- Homme/femme au foyer, sans revenus
- Etudiant(e)
- Pensionné(e)
- Invalidité
- Je ne sais pas
- Autre

En tenant compte de tous les revenus de votre ménage, dans quelle mesure parvenez-vous à joindre les deux bouts à la fin de chaque mois ? *

Veuillez sélectionner une réponse ci-dessous.
Veuillez sélectionner une seule des propositions suivantes :

- Très facilement
- Facilement
- Assez facilement
- Avec quelque difficulté
- Avec difficulté
- Avec grande difficulté
- Je ne sais pas

C'est à dire, dans quelle mesure est-ce que les revenus de votre ménage suffisent pour couvrir toutes vos dépenses chaque mois?

Concernant votre problème de santé

Depuis combien de temps approximativement avez-vous reçu le diagnostic de trouble psychotique? *

Veuillez sélectionner une réponse ci-dessous.
Veuillez sélectionner une seule des propositions suivantes :

- Moins de 6 mois
- 6 mois à 2 ans
- 2 à 5 ans
- 5 à 10 ans
- Plus de 10 ans
- Je ne sais pas

Indiquez approximativement le nombre d'années: *

Répondre à cette question seulement si les conditions suivantes sont réunies :
La réponse était 'Plus de 10 ans ' à la question ' [D13]' (Depuis combien de temps approximativement avez-vous reçu le diagnostic de trouble psychotique?)
Seuls des nombres peuvent être entrés dans ce champ.
Votre réponse doit être comprise entre 10 et 100
Veuillez écrire votre réponse ici :

-

Combien de temps est passé entre vos premiers symptômes psychotiques et le premier contact avec un psychiatre ? *

Veuillez sélectionner une réponse ci-dessous.
Veuillez sélectionner une seule des propositions suivantes :

- Moins de 1 mois
- Entre 1 mois et 6 mois
- Entre 6 mois et 1 an
- Entre 1 an et 2 ans
- Plus de 2 ans
- Je ne sais pas

Combien de temps est passé entre votre premier rendez-vous médical pour le trouble psychotique et le diagnostic? *

Veuillez sélectionner une réponse ci-dessous.
Veuillez sélectionner une seule des propositions suivantes :

- Moins de 1 mois
- Entre 1 mois et 6 mois
- Entre 6 mois et 1 an
- Entre 1 an et 2 ans
- Plus que 2 ans
- Je ne sais pas

Quel(s) diagnostic(s) avez-vous reçu(s) ? *

Répondre à cette question seulement si les conditions suivantes sont réunies :
((D13.NAOK != "A7"))
Cochez tout ce qui s’applique.

Veuillez choisir toutes les réponses qui conviennent :

- Schizophrénie
- Trouble schizo-affectif
- Trouble schizophréniforme
- Trouble délirant
- Trouble psychotique bref
- Trouble psychotique partagé
- Trouble psychotique induit par une substance
- Trouble psychotique dû à une autre affection médicale
- Trouble psychotique non spécifié
- Trouble bipolaire avec caractéristiques psychotiques
- Je ne sais pas
- Je ne veux pas répondre
- Autre:

Merci pour votre participation.

Malheureusement, vous ne rentrez pas dans les conditions pour pouvoir poursuivre cette étude, car nous n'incluons pas les troubles psychotiques dû à une autre affection médicale.

Répondre à cette question seulement si les conditions suivantes sont réunies :
((DSD1_A1.NAOK != "Y" and DSD1_A11.NAOK != "Y" and DSD1_A12.NAOK != "Y" and DSD1_A2.NAOK != "Y" and DSD1_A3.NAOK != "Y" and DSD1_A4.NAOK != "Y" and DSD1_A5.NAOK != "Y" and DSD1_A6.NAOK != "Y" and DSD1_A7.NAOK != "Y" and DSD1_A8.NAOK == "Y" and DSD1_A9.NAOK != "Y"))

Avez-vous déjà été hospitalisé(e) à cause du trouble psychotique? Considérez uniquement les hospitalisations avec nuitée(s) *

Répondre à cette question seulement si les conditions suivantes sont réunies :
((DSD1_A1.NAOK == "Y" or DSD1_A11.NAOK == "Y" or DSD1_A12.NAOK == "Y" or DSD1_A2.NAOK == "Y" or DSD1_A3.NAOK == "Y" or DSD1_A4.NAOK == "Y" or DSD1_A5.NAOK == "Y" or DSD1_A6.NAOK == "Y" or DSD1_A7.NAOK == "Y" or DSD1_A9.NAOK == "Y" or DSD1_A10.NAOK == "Y"))
Veuillez sélectionner une réponse ci-dessous.
Veuillez sélectionner une seule des propositions suivantes :

- Oui
- Non
- Je ne sais pas

{if(((is_empty(DSD1_A1.NAOK)) and (is_empty(DSD1_A2.NAOK)) and (is_empty(DSD1_A3.NAOK)) and (is_empty(DSD1_A4.NAOK)) and (is_empty(DSD1_A5.NAOK)) and (is_empty(DSD1_A6.NAOK)) and (is_empty(DSD1_A7.NAOK)) and (is_empty(DSD1_A9.NAOK)) and

(is_empty(DSD1_A11.NAOK)) and (is_empty(DSD1_A12.NAOK)) and (DSD1_A8.NAOK == "Y")),'uitgesloten','in')}

Autres problèmes de santé

Avez-vous un ou plusieurs des problèmes de santé suivants pouvant-être associés au trouble psychotique ? *

Choisissez la réponse appropriée pour chaque élément :

	Oui	Non	Je ne sais pas
Trouble obsessionnel compulsif (TOC)			
Dépression			
Trouble bipolaire			
Trouble de la consommation d'alcool			
Trouble lié à l'usage de substances psychoactives / toxicomanie			
Trouble anxieux ou trouble de panique			
Trouble du stress post-traumatique (TSPT)			
Trouble de la personnalité			
Trouble du spectre de l'autisme			
Trouble déficit de l'attention avec ou sans hyperactivité (TDAH)			
Trouble du comportement alimentaire (p.ex. anorexie, boulimie)			

Avez-vous également d' autre(s) problème(s) de santé ? *

Veuillez sélectionner une réponse ci-dessous.
Veuillez sélectionner une seule des propositions suivantes :

- Oui
- Non
- Je ne sais pas

Quels autres problèmes de santé avez-vous ? <u>Vous pouvez cocher plusieurs cases</u>.

Veuillez également indiquer le nom précis de votre/vos problème(s) de santé si vous le(s) connaissez.
Si vous ne connaissez pas (encore) le diagnostic de votre autre problème de santé, cochez la case “pas de diagnostic connu“.

*

Répondre à cette question seulement si les conditions suivantes sont réunies :
La réponse était 'Oui' à la question ' [D14]' (Avez-vous également d' autre(s) problème(s) de santé ?)
Ajoutez un commentaire seulement si vous sélectionnez la réponse.
Veuillez choisir toutes les réponses qui conviennent et laissez un commentaire :

- Maladie du cœur ou des vaisseaux sanguins (p.ex. hypertension)
- Maladie du système respiratoire (p.ex. asthme)
- Maladie de l'appareil digestif (p.ex. syndrôme du colon irritable)
- Maladie de la peau
- Maladie des os, articulations, ou muscles (p.ex. arthrose, arthrite rhumatoïde)
- Autre trouble de santé mentale ou psychologique non mentionné dans la question précédente
- Trouble neurodéveloppemental (p.ex. retard de développement)
- Maladie du système nerveux (neurologique) (p.ex. démence, sclérose en plaques)
- Maladie du sang (hématologique) (p.ex. anémie)
- Maladie immunitaire (p.ex. allergies, maladies auto-inflammatoires)
- Maladie endocrinienne, nutritionnelle, ou métabolique (p.ex. diabète, cholestérol élevé, obésité, hypothyroïdisme)
- Maladie de l'œil
- Maladie de l’oreille ou du système vestibulaire
- Maladie des organes reproducteurs ou du système urinaire (p.ex. insuffisance rénale, endométriose)
- Troubles du sommeil (p.ex. apnée obstructive du sommeil)
- Maladie qui touche plusieurs systèmes (p.ex. psoriasis)
- Pas de diagnostic connu
- Je ne sais pas
- Autre :

Votre état de santé général

Certaines des questions suivantes pourraient ne pas être directement liées à votre situation. Cependant, elles nous aident à mieux comprendre votre contexte. Nous vous remercions de prendre le temps d'y répondre.

Nous aimerions connaître votre état de santé tel que vous le percevez. Veuillez cocher la case à côté de l'affirmation qui décrit le mieux votre santé <u>juste AVANT l'apparition de vos premiers symptômes psychotiques.</u>

Considérez comme "premiers symptômes psychotiques" les premières manifestations de votre trouble psychotique, telles qu'entendre des voix ou des bruits qui n'existent pas

selon les autres ou de voir des choses que les autres ne voient pas, avoir des pensées qui ne correspondent pas à la réalité perçue par les autres, ou avoir l'idée que quelqu'un cherche volontairement à vous nuire. Si vous ne savez pas quand vos symptômes ont commencé, veuillez à la place considérer le moment de votre premier contact avec les soins psychiatriques.

Votre mobilité avant vos premiers symptômes de trouble psychotique *

Veuillez sélectionner une réponse ci-dessous.
Veuillez sélectionner une seule des propositions suivantes :

- Je n'avais aucun problème pour me déplacer à pied.
- J'avais des problèmes légers pour me déplacer à pied.
- J'avais des problèmes modérés pour me déplacer à pied.
- J'avais des problèmes sévères pour me déplacer à pied.
- J'étais incapable de me déplacer à pied.
- Je ne sais pas.

Votre autonomie avant vos premiers symptômes de trouble psychotique *

Veuillez sélectionner une réponse ci-dessous.
Veuillez sélectionner une seule des propositions suivantes :

- Je n'avais aucun problème pour me laver ou m'habiller tout(e) seul(e).
- J'avais des problèmes légers pour me laver ou m'habiller tout(e) seul(e).
- J'avais des problèmes modérés pour me laver ou m'habiller tout(e) seul(e).
- J'avais des problèmes sévères pour me laver ou m'habiller tout(e) seul(e).
- J'étais incapable de me laver ou de m'habiller tout(e) seul(e).
- Je ne sais pas.

Vos activités courantes avant vos premiers symptômes de trouble psychotique (exemples: travail, études, faire des courses, faire le ménage, prendre des transports en commun, activités avec la famille ou voir des amis, loisirs) *

Veuillez sélectionner une réponse ci-dessous.
Veuillez sélectionner une seule des propositions suivantes :

- Je n'avais aucun problème pour accomplir mes activités courantes.
- J'avais des problèmes légers pour accomplir mes activités courantes.
- J'avais des problèmes modérés pour accomplir mes activités courantes.
- J'avais des problèmes sévères pour accomplir mes activités courantes.
- J'étais incapable d'accomplir mes activités courantes.
- Je ne sais pas.

Votre douleur ou gêne avant vos premiers symptômes de trouble psychotique *

Veuillez sélectionner une réponse ci-dessous.
Veuillez sélectionner une seule des propositions suivantes :

- Je n'avais ni douleur ni gêne.
- J'avais des douleurs ou une gêne légère(s).
- J'avais des douleurs ou une gêne modérée(s).
- J'avais des douleurs ou une gêne sévère(s).
- J'avais des douleurs ou une gêne extrême(s).
- Je ne sais pas.

Votre anxiété ou dépression avant vos premiers symptômes de trouble psychotique *

Veuillez sélectionner une réponse ci-dessous.
Veuillez sélectionner une seule des propositions suivantes :

- Je n'étais ni anxieux(se), ni déprimé(e).
- J'étais légèrement anxieux(se) ou déprimé(e).
- J'étais modérément anxieux(se) ou déprimé(e).
- J'étais sévèrement anxieux(se) ou déprimé(e).
- J'étais extrêmement anxieux(se) ou déprimé(e).
- Je ne sais pas.

Nous aimerions savoir dans quelle mesure votre santé était bonne ou mauvaise AVANT vos premiers symptômes de trouble psychotique sur une échelle de 0 à 100.

100 correspond à la meilleure santé que vous puissiez imaginer.
0 correspond à la pire santé que vous puissiez imaginer.

Veuillez entrer un nombre entre 0 et 100 afin d'indiquer votre état de santé AVANT vos premiers symptômes de trouble psychotique.

Votre réponse doit être comprise entre 0 et 100
Seul un nombre entier peut être inscrit dans ce champ.
Veuillez écrire votre réponse ici :

-

Nous aimerions connaitre votre état de santé actuel, tel que vous le vivez aujourd'hui. Veuillez cocher la case à côté de l'affirmation qui décrit le mieux votre santé AUJOURD'HUI.

Votre mobilité aujourd'hui *

Veuillez sélectionner une réponse ci-dessous.
Veuillez sélectionner une seule des propositions suivantes :

- Je n’ai aucun problème pour me déplacer à pied.
- J’ai des problèmes légers pour me déplacer à pied.
- J’ai des problèmes modérés pour me déplacer à pied.
- J’ai des problèmes sévères pour me déplacer à pied.
- Je suis incapable de me déplacer à pied.
- Je ne sais pas.

Votre autonomie aujourd'hui *

Veuillez sélectionner une réponse ci-dessous.
Veuillez sélectionner une seule des propositions suivantes :

- Je n’ai aucun problème pour me laver ou m’habiller tout(e) seul(e).
- J’ai des problèmes légers pour me laver ou m’habiller tout(e) seul(e).
- J’ai des problèmes modérés pour me laver ou m’habiller tout(e) seul(e).
- J’ai des problèmes sévères pour me laver ou m’habiller tout(e) seul(e).
- Je suis incapable de me laver ou de m’habiller tout(e) seul(e).
- Je ne sais pas.

Vos activités courantes aujourd'hui (exemples: travail, études, faire des couses, faire le ménage, prendre des transports en commun, activités avec la famille ou voir des amis, loisirs)

*****

Veuillez sélectionner une réponse ci-dessous.
Veuillez sélectionner une seule des propositions suivantes :

- Je n’ai aucun problème pour accomplir mes activités courantes.
- J’ai des problèmes légers pour accomplir mes activités courantes.
- J’ai des problèmes modérés pour accomplir mes activités courantes.
- J’ai des problèmes sévères pour accomplir mes activités courantes.
- Je suis incapable d’accomplir mes activités courantes.
- Je ne sais pas.

Votre douleur ou gêne aujourd'hui *

Veuillez sélectionner une réponse ci-dessous.
Veuillez sélectionner une seule des propositions suivantes :

- Je n’ai ni douleur ni gêne.
- J’ai des douleurs ou une gêne légère(s).
- J’ai des douleurs ou une gêne modérée(s).
- J’ai des douleurs ou une gêne sévère(s).
- J’ai des douleurs ou une gêne extrême(s).
- Je ne sais pas.

Votre anxiété ou dépression aujourd'hui *

Veuillez sélectionner une réponse ci-dessous.
Veuillez sélectionner une seule des propositions suivantes :

- Je ne suis ni anxieux(se), ni déprimé(e).
- Je suis légèrement anxieux(se) ou déprimé(e).
- Je suis modérément anxieux(se) ou déprimé(e).
- Je suis sévèrement anxieux(se) ou déprimé(e).
- Je suis extrêmement anxieux(se) ou déprimé(e).
- Je ne sais pas.

Nous aimerions savoir dans quelle mesure votre santé est bonne ou mauvaise AUJOURD'HUI sur une échelle de 0 à 100.

**100 correspond à <u>la meilleure santé</u> que vous puissiez imaginer.
0 correspond à <u>la pire santé</u> que vous puissiez imaginer.**

Veuillez entrer un nombre entre 0 et 100 afin d'indiquer votre état de santé AUJOURD'HUI.

Votre réponse doit être comprise entre 0 et 100
Seul un nombre entier peut être inscrit dans ce champ.
Veuillez écrire votre réponse ici :

-

Traitements liés au trouble psychotique

Avez-vous reçu un traitement lié à votre trouble psychotique <u>dans les 5 dernières années</u>? *

Veuillez sélectionner une réponse ci-dessous.
Veuillez sélectionner une seule des propositions suivantes :

- Oui
- Non
- Je ne sais pas

Quels traitements avez-vous reçus pour le trouble psychotique <u>dans les 5 dernières années</u> ?

*****

Répondre à cette question seulement si les conditions suivantes sont réunies :
La réponse était 'Oui' à la question ' [HC2]' (Avez-vous reçu un traitement lié à votre trouble psychotique dans les 5 dernières années?)
Cochez tout ce qui s'applique.
Veuillez choisir toutes les réponses qui conviennent :

- Médicaments antipsychotiques « atypiques » (de 2ème génération) (p.ex. Aripiprazole, Abilify, Sycrest, Reagila, Olanzapine, Zypadhera, Invega, Xeplion, Trevicta, Quétiapine, Risperidone, Serdolect)
- Médicaments antipsychotiques « classiques » (de 1ère génération) (p.ex. Clopixol, Haldol, Nozinan, Fluanxol, Etumine, Deanxit)
- Clozapine (p.ex. Clozapine Sandoz, Leponex)
- Antidépresseurs utilisés pour traiter la dépression, le trouble obsessionel compulsif, ou trouble de panique (p.ex. Citalopram, Escitalopram, Fluoxetine, Sertraline, Edronax, Redomex, Duloxetine, Venlafaxine)
- Tranquillisants (p.ex. Diazepam, Valium, Lormetazepam, Lorazepam)
- Psychothérapie (p.ex. thérapie cognitivo-comportementale, psychoéducation, etc)
- Electroconvulsivothérapie (ECT)
- Autre stimulation cérébrale non invasive : stimulation transcrânienne à courant direct (tDCS), ou la stimulation magnétique transcrânienne répétitive (rTMS).
- Stimulation cérébrale profonde
- Exercice physique
- Soutien par les pairs
- Je ne sais pas
- Autre:

Quel(s) effet(s) indésirable(s) avez-vous eu pendant ou après le traitement du trouble psychotique <u>dans les 5 dernières années</u>?

*

Répondre à cette question seulement si les conditions suivantes sont réunies :
((HC2.NAOK == "A1"))
Cochez tout ce qui s'applique.
Veuillez choisir toutes les réponses qui conviennent :

- Prise de poids
- Hyperglycémie (taux de sucre élevé) ou diabète
- Taux élevé de cholestérol (excès de graisses sanguines)
- Manque de globules blancs
- Troubles du mouvement
- Agitation, difficulté à rester immobile, besoin de bouger
- Contractions musculaires involontaires, et parfois douloureuses
- Tremblements et/ou rigidité faciale
- Mouvements involontaires du visage, comme mâchonner, tirer la langue ou cligner des yeux
- Fatigue ou somnolence
- Vertiges
- Sécheresse de la bouche ou des yeux
- Nausées
- Constipation
- Myocardite (inflammation du muscle cardiaque)
- Dysfonction sexuelle (p.ex. baisse de la libido)
- Problèmes de mémoire ou "brouillard mental"

- Émoussement affectif (manque de motivation, manque d'intérêt)
- Aucun effet indésirable
- Autre:

Indiquez dans quelle mesure chacun des effets indésirables était pénible pour vous. *

Répondre à cette question seulement si les conditions suivantes sont réunies :
La réponse était 'Oui' à la question ' [HC2]' (Avez-vous reçu un traitement lié à votre trouble psychotique dans les 5 dernières années?) *et* La réponse n'était PAS 'Aucun effet indésirable' à la question ' [HC4]' (Quel(s) effet(s) indésirable(s) avez-vous eu pendant ou après le traitement du trouble psychotique dans les 5 dernières années?)
Choisissez la réponse appropriée pour chaque élément :

	Pas du tout pénible	Légèrement pénible	Modérément pénible	Très pénible	Extrêmement pénible	Je ne sais pas
**Prise de poids**						
**Hyperglycémie (taux de sucre élevé) ou diabete**						
**Taux élevé de cholestérol (excès de graisses sanguines)**						
**Manque de globules blancs**						
**Agitation, difficulté à rester immobile, besoin de bouger**						
**Contractions musculaires involontaires et parfois douloureuses**						
**Tremblements et/ou rigidité faciale**						
**Mouvements involontaires du visage, comme mâchonner, tirer la langue ou clinger des yeux**						
**Fatigue ou somnolence**						
**Vertiges**						

	Pas du tout pénible	Légèrement pénible	Modérément pénible	Très pénible	Extrêmement pénible	Je ne sais pas
**Sécheresse de la bouche ou des yeux**						
**Naussées**						
**Constipation**						
**Myocardite (inflammation du muscle cardiaque)**						
**Dysfonction sexuelle (p.ex. baisse de la libido)**						
**Problèmes de mémoire ou "brouillard mental"**						
**Emoussement affectif (manque de motivation, manque d'intérêt)**						
**Aucun effet indésirable**						

Par pénible nous entendons: désagréable, fatigant ou contraignant, qui cause une gêne ou un inconfort physique ou mental.

<u>En dehors des effets indésirables</u>, dans quelle mesure le traitement que vous avez reçu pour le trouble psychotique au cours des 5 dernières années, a-t-il été pénible pour vous? Par exemple, en raison de l'administration par injection, du besoin de se rendre à l'hôpital pour recevoir le traitement, d'un horaire de prise contraignant, de la nécessité de conserver le médicament au frigo, etc. *

Répondre à cette question seulement si les conditions suivantes sont réunies :
((HC2.NAOK == "A1"))
Veuillez sélectionner une réponse ci-dessous.
Veuillez sélectionner une seule des propositions suivantes :

- Pas du tout pénible
- Légèrement pénible
- Modérément pénible
- Très pénible
- Extrêmement pénible
- Je ne sais pas

Symptôme(s) lié(s) à votre trouble psychotique

<u>Depuis que vous êtes suivi(e) ou traité(e) pour le trouble psychotique</u>, quels symptômes parmi les suivants avez-vous ressenti et dans quelle mesure étaient-ils pénibles pour vous ? Ne tenez pas compte des effets indésirables du traitement. *

Choisissez la réponse appropriée pour chaque élément :

	Je n'ai pas eu ce symptôme	Pas du tout pénible	Légèrement pénible	Modérément pénible	Très pénible	Extrêmement pénible	Je ne sais pas
L'impression que l'on dise à la télévision ou que l'on écrive dans les journaux des choses spécialement pour vous, l'impression que l'on cherche volontairement à vous nuire, l'impression qu'il y a un complot dirigé contre vous, l'impression d'entendre vos pensées répétées comme par un écho, ou d'autres perceptions altérées de la réalité.							
Entendre, voir ou ressentir quelque chose qui n'est pas là. Par exemple, entendre une ou plusieurs voix alors que							

	Je n'ai pas eu ce symptôme	Pas du tout pénible	Légèrement pénible	Modérément pénible	Très pénible	Extrêmement pénible	Je ne sais pas
vous êtes tout(e) seul(e) ; voir des objets, des personnes ou des animaux que les autres personnes ne voient pas.							
Sensation d’influence, de contrôle, ou de passivité. Par exemple, l'impression d'avoir dans votre tête des pensées que vous ne reconnaissez pas comme les vôtres. L'impression d'être sous le contrôle d’une force ou d’un pouvoir extérieur à vous-même.							
Désorganisation de la pensée, comme le sentiment d’avoir l’esprit vide, ou l'impression que vos pensées ont été enlevées ou extraites de votre tête, ou d'avoir dans votre tête des pensées que vous ne reconnaissez							

	Je n'ai pas eu ce symptôme	Pas du tout pénible	Légèrement pénible	Modérément pénible	Très pénible	Extrêmement pénible	Je ne sais pas
pas comme les vôtres							
Désorganisation du comportement. Par exemple, le sentiment que vous négligez votre apparence ou votre hygiène personnelle, ou le sentiment que vous n’arrivez jamais à terminer ce que vous faites.							
Incapacité à éprouver de l’intérêt ou du plaisir, manque de motivation ou d’enthousiasme, sentiment que vos émotions sont émoussées.							
Isolement social, ou le sentiment que vous n’éprouvez pas d’intérêt à être avec d’autres personnes.							
Agitation, ou sentiment de tension							
Problèmes de concentration							
Fatigue ou épuisement							

	Je n'ai pas eu ce symptôme	Pas du tout pénible	Légèrement pénible	Modérément pénible	Très pénible	Extrêmement pénible	Je ne sais pas
Peur ou anxiété							
Sentiment d'abattement, de déprime, ou de tristesse							
Stress							
Comportements ou envies de se faire du mal ou de se blesser							
L'impression de n'avoir plus envie de vivre							
Stigmatisation ou sentiment de discrimination							
Perte de confiance en soi							
Autre							

Dans quelle mesure est-ce que le trouble psychotique a interféré avec votre vie sexuelle ? *

Veuillez sélectionner une réponse ci-dessous.
Veuillez sélectionner une seule des propositions suivantes :

- Pas du tout
- Légèrement
- Modérément
- Beaucoup
- Extrêmement
- Je ne sais pas
- Ne s'applique pas
- Je ne veux pas répondre

Dans quelle mesure le trouble psychotique a-t-il interféré avec votre vie reproductive, c'est-à-dire avec votre possibilité ou décision de concevoir naturellement des enfants ou de mener à terme une grossesse?

Par exemple: FIV (Fécondation in Vitro), dépistage génétique, don de

sperme/ovocytes, nécessité de retarder une grossesse, ou renoncé à avoir des enfants biologiques à cause du trouble psychotique. *

Veuillez sélectionner une réponse ci-dessous.
Veuillez sélectionner une seule des propositions suivantes :

- Pas du tout
- Légèrement
- Modérément
- Beaucoup
- Extrêmement
- Je ne sais pas
- Ne s’applique pas (p.ex. vous n'avez pas encore envisagé d'avoir des enfants, ou vous n'étiez pas touché par le trouble psychotique quand vous étiez en âge d’avoir des enfants)
- Je ne veux pas répondre

Prise en charge médicale du trouble psychotique

Par 'prise en charge' nous entendons l'ensemble des rendez-vous, traitements, et accompagnements proposés par les professionnels de santé pour gérer et améliorer votre état de santé en lien avec le trouble psychotique.

Considérez-vous que votre prise en charge a été bien <u>organisée et planifiée</u> au fil du temps? *

Veuillez sélectionner une réponse ci-dessous.
Veuillez sélectionner une seule des propositions suivantes :

- Toujours
- Souvent
- Parfois
- Rarement
- Jamais
- Je ne sais pas

Avez-vous un ou plusieurs professionnels de santé qui sont responsables de la <u>coordination</u> des différents services médicaux dont vous avez besoin ?

*****

Veuillez sélectionner une réponse ci-dessous.
Veuillez sélectionner une seule des propositions suivantes :

- Oui

- Non
- Je ne sais pas
- Ne s’applique pas : le suivi se fait uniquement dans un service, sans besoin de coordination

Par exemple, un médecin ou une infirmière de référence qui vous suit ou vous oriente, et facilite la communication entre les différents spécialistes ou services que vous consultez.

Dans quelle mesure voyez-vous le(s) <u>même(s)</u> professionnel(s) de santé pendant vos rendez-vous ou examens réguliers ? *

Veuillez sélectionner une réponse ci-dessous.
Veuillez sélectionner une seule des propositions suivantes :

- Toujours
- Souvent
- Parfois
- Rarement
- Jamais
- Je ne sais pas

Dans quelle mesure recevez-vous suffisamment d'informations <u>utiles et compréhensibles</u> de la part de vos professionnels de santé?

*****

Veuillez sélectionner une réponse ci-dessous.
Veuillez sélectionner une seule des propositions suivantes :

- Toujours
- Souvent
- Parfois
- Rarement
- Jamais
- Je ne sais pas

Par utiles, nous entendons des informations à jour et cohérentes qui vous aident à mieux comprendre et gérer votre trouble psychotique.

Êtes-vous impliqué(e) autant que vous le souhaitez dans les décisions concernant vos soins ou votre traitement ? *

Veuillez sélectionner une réponse ci-dessous.
Veuillez sélectionner une seule des propositions suivantes :

- Oui, certainement
- Oui, dans une certaine mesure

- Non, pas vraiment
- Non, certainement pas
- Non, mais je ne voulais de toute façon pas être impliqué(e)
- Pas applicable: pas de décisions prises à propos du traitement
- Je ne sais pas

Accès aux soins de santé

<u>Au cours des 12 derniers mois</u>, y a-t-il des soins pour le trouble psychotique que vous n'avez pas obtenus alors que vous en auriez eu besoin ?
Il peut s'agir d'une consultation, d'un traitement, d'analyses médicales, de rééducation, ou autre. *

Veuillez sélectionner une réponse ci-dessous.
Veuillez sélectionner une seule des propositions suivantes :

- Oui
- Non
- Je ne sais pas

Pour quelle(s) raison(s) principale(s) n'avez-vous pas reçu les soins dont vous aviez besoin?

*

Répondre à cette question seulement si les conditions suivantes sont réunies :
La réponse était 'Oui' à la question ' [HC12]' (Au cours des 12 derniers mois, y a-t-il des soins pour le trouble psychotique que vous n'avez pas obtenus alors que vous en auriez eu besoin ? Il peut s'agir d'une consultation, d'un traitement, d'analyses médicales, de rééducation, ou autre.)
Cochez tout ce qui s'applique.
Veuillez choisir toutes les réponses qui conviennent :

- Distance entre le domicile et le lieu de soins
- Problème de transport (par exemple pas de transport en commun à proximité, pas de véhicule personnel, impossibilité de conduire votre propre véhicule, personne pour vous emmener sur le lieu de vos soins)
- Manque de personnel compétent pour vous administrer les soins dont vous avez besoin
- Délais d'attente très longs
- Difficultés pour payer les soins
- Peur des examens médicaux, de l'hôpital, ou d'autre chose
- Manque de temps à cause du travail, des enfants, ou autre
- Manque d'information
- Difficultés liées à la langue
- Autre:

Impact du trouble psychotique sur l'éducation et sur le travail

Dans quelle mesure votre scolarité ou vos études ont-elles été affectées par le trouble psychotique, en termes de présence, de performance, de réussite, ou de choix d'études? *

Veuillez sélectionner une réponse ci-dessous.
Veuillez sélectionner une seule des propositions suivantes :

- Pas du tout
- Légèrement
- Modérément
- Beaucoup
- Extrêmement
- Je ne sais pas
- Ne s'applique pas: le trouble psychotique est survenu après que la scolarité/les études soient terminées

<u>En raison du trouble psychotique</u>, avez-vous diminué votre temps de travail? *

Répondre à cette question seulement si les conditions suivantes sont réunies :
((D10.NAOK == "A1"))
Veuillez sélectionner une réponse ci-dessous.
Veuillez sélectionner une seule des propositions suivantes :

- Oui, je travaille moins qu'avant en raison de mon trouble psychotique
- Oui, j'ai arrêté de travailler en raison de mon trouble psychotique
- Non, le trouble psychotique n'a pas eu d'impact sur mon temps de travail
- Je ne sais pas
- Autre

Combien de temps, approximativement, avez-vous été en incapacité de travailler en raison de votre trouble psychotique? *

Veuillez sélectionner une réponse ci-dessous.
Veuillez sélectionner une seule des propositions suivantes :

- Pendant moins de 1 mois
- Pendant 1 à 3 mois
- Pendant 4 mois à 1 an
- Pendant plus de 1 an
- Je ne sais pas

Veuillez donner une estimation approximative du TOTAL de tous les arrêts de travail ou congés maladie, qu'ils soient consécutifs ou non. Par exemple : si vous avez été en arrêt pendant 1 mois, puis, plus tard, pendant 3 mois cocher « pendant 4 mois à 1 an ».

Veuillez indiquez approximativement le nombre d'années: *

Répondre à cette question seulement si les conditions suivantes sont réunies :
La réponse était 'Pendant plus de 1 an ' à la question ' [S3]' (Combien de temps, approximativement, avez-vous été en incapacité de travailler en raison de votre trouble psychotique?)
Seuls des nombres peuvent être entrés dans ce champ.
Veuillez écrire votre réponse ici :

-

La prochaine question concerne les sept derniers jours, sans inclure aujourd'hui. *

Répondre à cette question seulement si les conditions suivantes sont réunies :
La réponse était 'Non, le trouble psychotique n'a pas eu d'impact sur mon temps de travail' *ou* 'Oui, je travaille moins qu'avant en raison de mon trouble psychotique' à la question ' [S2]' (En raison du trouble psychotique, avez-vous diminué votre temps de travail?)

<u>Au cours des sept derniers jours</u>, dans quelle mesure le trouble psychotique a-t-il affecté votre productivité <u>pendant que vous travailliez</u> ?

Pensez aux jours où vous avez été limité(e) dans la quantité ou le type de travail que vous pouviez effectuer, aux jours où vous avez accompli moins que vous ne l'auriez souhaité ou aux jours où vous n'avez pas pu faire votre travail avec autant de soin que d'habitude. Si le trouble psychotique n'a que peu affecté votre travail, choisissez un chiffre faible. Choisissez un chiffre élevé si le trouble psychotique a beaucoup affecté votre travail.

*****

Répondre à cette question seulement si les conditions suivantes sont réunies :
La réponse était 'Non, le trouble psychotique n'a pas eu d'impact sur mon temps de travail' *ou* 'Oui, je travaille moins qu'avant en raison de mon trouble psychotique' à la question ' [S2]' (En raison du trouble psychotique, avez-vous diminué votre temps de travail?)
Chaque entrée doit être entre 0 et 10
Seuls des nombres entiers peuvent être inscrits dans ces champs.
Veuillez écrire votre(vos) réponse(s) ici :

- |

Considérez uniquement dans quelle mesure le trouble psychotique a affecté votre productivité pendant que vous travailliez.

Cliquez sur le curseur pour le faire glisser sur la valeur choisie

Conséquences financières

Dans quelle mesure votre trouble psychotique ou votre traitement médical vous a-t-il causé des difficultés financières? *

Veuillez sélectionner une réponse ci-dessous.
Veuillez sélectionner une seule des propositions suivantes :

- Pas du tout
- Légèrement
- Modérément
- Beaucoup
- Extrêmement
- Je ne sais pas

Impact sur les relations sociales et besoins de soutien

Dans quelle mesure votre trouble psychotique interfère-t-il avec vos relations avec la famille, votre partenaire, vos amis, ou voisins ? *

Veuillez sélectionner une réponse ci-dessous.
Veuillez sélectionner une seule des propositions suivantes :

- Pas du tout
- Légèrement
- Modérément
- Beaucoup
- Extrêmement
- Je ne sais pas

À cause du trouble psychotique, de quels types de soutien supplémentaire avez-vous eu besoin, <u>sans y être parvenu jusqu’à présent</u>?

*****

Cochez tout ce qui s’applique.
Veuillez choisir toutes les réponses qui conviennent :

- Aucun soutien supplémentaire
- Aide logistique (par exemple: nettoyage, cuisine, courses, transport, garde d'enfants)

- Parler à d’autres patients atteints d'un trouble psychotique
- Soutien psychologique
- Soutien médical
- Aide administrative ou sociale
- Soutien pour l'adaptation de l'environnement, ou matériel adapté
- Soutien pour le retour au travail
- Soutien scolaire ou pour les études
- Accompagnement spirituel ou religieux
- Je ne sais pas
- Autre:

Priorité des besoins

Selon vous, quels aspects de votre trouble psychotique ou de sa prise en charge devraient être améliorés en priorité ? Veuillez évaluer chaque aspect selon l’importance que vous lui accordez. *

Choisissez la réponse appropriée pour chaque élément :

	Pas du tout prioritaire	Légèrement prioritaire	Modérément prioritaire	Très prioritaire	Extrêmement prioritaire	Je ne sais pas
L’impact du trouble psychotique sur la qualité de vie générale (impact sur la mobilité, l'autonomie, la capacité à vous habiller et laver seul, la gêne ou douleur, l'anxiété ou la dépression; années vécues avec une incapacité)						
L’impact sur votre santé physique (symptômes physiques, gêne ou douleur liées au à le touble psychotique)						
L’impact sur votre santé psychologique (symptômes psychologiques, anxiété, ou dépression liées au à le trouble psychotique)						

	Pas du tout prioritaire	Légèrement prioritaire	Modérément prioritaire	Très prioritaire	Extrêmement prioritaire	Je ne sais pas
L'impact sur votre autonomie (impact sur votre mobilité, sur votre capacité à vous laver et habiller seul(e) ; capacité à accomplir vos activités courantes comme le travail, études, travaux ménagers, activités familiales, ou loisirs)						
L'impact sur votre vie sexuelle et reproductive						
L'impact sur l'espérance de vie						
L'impact sur votre scolarité ou vos études						
L'impact sur votre travail						
L'impact financier						
L'impact sur votre vie sociale (relations avec la famille, votre partenaire, ou vos amis ; besoin de soutien supplémentaire-administratif, social, etc)						
La qualité de votre prise en charge (la rapidité du diagnostic, la coordination et la continuité des soins, les informations reçues par les professionnels de santé, l'implication dans les décisions concernant vos soins ou traitements)						
L'accès aux soins de santé (obtenir des soins lorsque vous en avez						

	Pas du tout prioritaire	Légèrement prioritaire	Modérément prioritaire	Très prioritaire	Extrêmement prioritaire	Je ne sais pas
besoin, la disponibilité du traitement)						
Les connaissances scientifiques (par exemple en termes de diagnostic, de traitement, ou de pronostic)						
L'efficacité du traitement (capacité du traitement à contrôler les symptômes et améliorer la qualité de vie)						
La pénibilité du traitement (en termes d'effets indésirables ou en termes de logistique/administration du traitement)						

Autre besoins importants

Souhaitez-vous nous faire part d'un ou de plusieurs autres besoins importants que vous n'avez pas pu exprimer dans le questionnaire ? *

Veuillez sélectionner une seule des propositions suivantes :

- Oui
- Non

Quels besoins n'ont pas été abordés dans le questionnaire ?

Répondre à cette question seulement si les conditions suivantes sont réunies :
La réponse était 'Oui' à la question ' [G1]' (Souhaitez-vous nous faire part d'un ou de plusieurs autres besoins importants que vous n'avez pas pu exprimer dans le questionnaire ?)
Veuillez écrire votre réponse ici :

Impact sur les aidants proches

En tant que personne remplissant le questionnaire pour quelqu'un atteint d'un trouble psychotique: vous considérez-vous comme « aidant proche » ?

C'est-à-dire venant en aide à cette personne en raison de son trouble psychotique, de manière régulière et à titre non professionnel. Par exemple, un parent aidant son enfant, un membre de la famille ou un ami aidant un proche.

*****

Veuillez sélectionner une seule des propositions suivantes :

- Oui
- Non

Par rapport à la personne que vous aidez, vous êtes : *

Répondre à cette question seulement si les conditions suivantes sont réunies :
La réponse était 'Oui' à la question ' [CG1]' (En tant que personne remplissant le questionnaire pour quelqu'un atteint d'un trouble psychotique: vous considérez-vous comme « aidant proche » ? C'est-à-dire venant en aide à cette personne en raison de son trouble psychotique, de manière régulière et à titre non professionnel. Par exemple, un parent aidant son enfant, un membre de la famille ou un ami aidant un proche.)
Veuillez sélectionner une réponse ci-dessous.
Veuillez sélectionner une seule des propositions suivantes :

- Un membre de la famille (par exemple, parent, enfant, grand-parent…)
- Une relation proche (par exemple, ami, voisin…)
- Autre

A quel genre vous identifiez-vous ?

Répondre à cette question seulement si les conditions suivantes sont réunies :
La réponse était 'Oui' à la question ' [CG1]' (En tant que personne remplissant le questionnaire pour quelqu'un atteint d'un trouble psychotique: vous considérez-vous comme « aidant proche » ? C'est-à-dire venant en aide à cette personne en raison de son trouble psychotique, de manière régulière et à titre non professionnel. Par exemple, un parent aidant son enfant, un membre de la famille ou un ami aidant un proche.)
Veuillez sélectionner une réponse ci-dessous.
Veuillez sélectionner une seule des propositions suivantes :

- Homme
- Femme
- Autre

Quelle est votre année de naissance ? *

Répondre à cette question seulement si les conditions suivantes sont réunies :
La réponse était 'Oui' à la question ' [CG1]' (En tant que personne remplissant le questionnaire pour quelqu'un atteint d'un trouble psychotique: vous considérez-vous comme « aidant proche » ? C'est-à-dire venant en aide à cette personne en raison de son trouble psychotique, de manière régulière et à titre non professionnel. Par exemple, un parent aidant son enfant, un membre de la famille ou un ami aidant un proche.)
Seuls des nombres peuvent être entrés dans ce champ.
Votre réponse doit être d'au moins 1920
Veuillez écrire votre réponse ici :

-

La semaine dernière, combien de temps avez-vous passé à aider la personne dans des activités qu'elle aurait normalement faites elle-même si elle était en bonne santé ? Par exemple dans les soins personnels (comme s'habiller/se déshabiller, aller aux toilettes…), les tâches ménagères (comme préparer les repas, nettoyer…) ou le soutien pratique (comme l'accompagner à l'extérieur à des rendez-vous médicaux ou autres…).

Répondre à cette question seulement si les conditions suivantes sont réunies :
La réponse était 'Oui' à la question ' [CG1]' (En tant que personne remplissant le questionnaire pour quelqu'un atteint d'un trouble psychotique: vous considérez-vous comme « aidant proche » ? C'est-à-dire venant en aide à cette personne en raison de son trouble psychotique, de manière régulière et à titre non professionnel. Par exemple, un parent aidant son enfant, un membre de la famille ou un ami aidant un proche.)
Seuls des nombres peuvent être entrés dans ce champ.
Veuillez écrire votre réponse ici :

- heures la semaine dernière.

Selon vous, dans quelle mesure <u>votre santé physique et/ou psychologique</u> est impactée en raison de votre rôle d'aidant proche? *

Répondre à cette question seulement si les conditions suivantes sont réunies :
La réponse était 'Oui' à la question ' [CG1]' (En tant que personne remplissant le questionnaire pour quelqu'un atteint d'un trouble psychotique: vous considérez-vous comme « aidant proche » ? C'est-à-dire venant en aide à cette personne en raison de son trouble psychotique, de manière régulière et à titre non professionnel. Par exemple, un parent aidant son enfant, un membre de la famille ou un ami aidant un proche.)
Veuillez sélectionner une réponse ci-dessous.
Veuillez sélectionner une seule des propositions suivantes :

- Pas du tout
- Légèrement
- Modérément
- Beaucoup
- Extrêmement

- Je ne sais pas

Selon vous, dans quelle mesure votre vie sociale est impactée en raison de votre rôle d'aidant proche? *

Répondre à cette question seulement si les conditions suivantes sont réunies :
La réponse était 'Oui' à la question ' [CG1]' (En tant que personne remplissant le questionnaire pour quelqu'un atteint d'un trouble psychotique: vous considérez-vous comme « aidant proche » ? C'est-à-dire venant en aide à cette personne en raison de son trouble psychotique, de manière régulière et à titre non professionnel. Par exemple, un parent aidant son enfant, un membre de la famille ou un ami aidant un proche.)
Veuillez sélectionner une réponse ci-dessous.
Veuillez sélectionner une seule des propositions suivantes :

- Pas du tout
- Légèrement
- Modérément
- Beaucoup
- Extrêmement
- Je ne sais pas

Poursuite de l'étude

Nous aimerions en savoir plus sur les besoins et les difficultés que vous rencontrez ou avez rencontrées dans le contexte de votre trouble psychotique. Si vous le souhaitez, vous pouvez participer à un entretien individuel (en ligne ou en face à face avec un chercheur) pour discuter plus en détail de vos expériences et de vos besoins. Cet entretien se déroulera de manière confidentielle et indépendamment des soins que vous recevez des prestataires de soins de santé. Il est prévu que 24 patients soient interrogés. Cet entretien individuel durera environ une heure et demie.

Souhaitez-vous participer à un entretien individuel?

*****

Veuillez sélectionner une seule des propositions suivantes :

- Oui
- Non

Si vous êtes intéressé(e) par l'entretien individuel, merci de nous indiquer vos coordonnées (numéro de téléphone et adresse mail) afin que nous puissions vous contacter. *

Répondre à cette question seulement si les conditions suivantes sont réunies :

La réponse était 'Oui' à la question ' [Follow]' (Nous aimerions en savoir plus sur les besoins et les difficultés que vous rencontrez ou avez rencontrées dans le contexte de votre trouble psychotique. Si vous le souhaitez, vous pouvez participer à un entretien individuel (en ligne ou en face à face avec un chercheur) pour discuter plus en détail de vos expériences et de vos besoins. Cet entretien se déroulera de manière confidentielle et indépendamment des soins que vous recevez des prestataires de soins de santé. Il est prévu que 24 patients soient interrogés. Cet entretien individuel durera environ une heure et demie. Souhaitez-vous participer à un entretien individuel?)
Les adresses e-mail doivent correspondre
Veuillez écrire votre(vos) réponse(s) ici :

- Adresse e-mail
- Confirmation de l'adresse e-mail
- Numéro de GSM

Merci beaucoup pour votre collaboration!

Un membre de l’équipe de recherche vous recontactera d’ici quelques semaines pour vous tenir informé(e) de votre éventuelle participation à un entretien.

Répondre à cette question seulement si les conditions suivantes sont réunies :
La réponse était 'Oui' à la question ' [Follow]' (Nous aimerions en savoir plus sur les besoins et les difficultés que vous rencontrez ou avez rencontrées dans le contexte de votre trouble psychotique. Si vous le souhaitez, vous pouvez participer à un entretien individuel (en ligne ou en face à face avec un chercheur) pour discuter plus en détail de vos expériences et de vos besoins. Cet entretien se déroulera de manière confidentielle et indépendamment des soins que vous recevez des prestataires de soins de santé. Il est prévu que 24 patients soient interrogés. Cet entretien individuel durera environ une heure et demie. Souhaitez-vous participer à un entretien individuel?)

Merci pour votre intérêt pour cette étude.

Les résultats de cette étude seront publiés sur le site Internet du KCE (www.kce.fgov.be) et sur le site de NEED (www.health-needs.eu) début 2026.

Veuillez noter que tous les résultats publiés concerneront uniquement des données agrégées, garantissant qu'aucune personne ne pourra être identifiée. La confidentialité et votre vie privée sont ainsi entièrement protégées.

Si remplir ce questionnaire ou réfléchir à votre trouble psychotique a suscité un inconfort ou une détresse émotionnelle, nous vous encourageons à rechercher du soutien. Vous pouvez contacter l'une de nos chercheuses, Margaux Sageot, au numéro 04 73 73 36 06. Vous pouvez également contacter votre médecin généraliste, un psychiatre ou un psychologue.

En cas d’urgence, vous pouvez appeler le 112. Si vous avez besoin de parler à quelqu'un dans l'anonymat et la confidentialité, vous pouvez appeler le 107.

Envoyer votre questionnaire.
Merci d’avoir complété ce questionnaire.
