# Supplementary material for: Refining the NEED (Needs Examination, Evaluation and Dissemination) Methods for Use in Psychotic Disorders in Belgium: Lessons From the Design Phase
Source: Health Expect. 2026 Aug 31;29(5):e70724. doi: 10.1111/hex.70724 (PMC13527729; doi:10.1111/hex.70724)
Supplement: Supplementary file 2 [file HEX-29-e70724-s002.pdf]

Appendix I: Recruitment Posters and Flyers Used in the Psychotic Disorders NEED Study (Dutch, French, and English Versions)

This appendix includes the posters and flyers used for participant recruitment, provided in both Dutch (NL) and French (FR). These illustrate the visual and linguistic considerations described in the manuscript. An English (ENG) translation has been prepared for the purpose of this publication; however, these materials were not used in the study and have not been validated with patients.

S70414

Trouble psychotique ?

Aidez-nous à mieux comprendre les besoins non rencontrés des personnes atteintes d'un trouble psychotique en participant à notre enquête.

Votre voix compte !

Conditions de participation:

- **Vous avez 18 ans ou plus**
- **Vous vivez en Belgique**
- **Vous comprenez le français ou le néerlandais**
- **Vous avez reçu un diagnostic de trouble psychotique (par ex. schizophrénie, trouble schizoaffectif, etc.)**

OU

- **Vous êtes le représentant légal d'une personne**
- **atteinte d'un trouble psychotique qui n'est pas en mesure de participer elle-même à l'enquête**

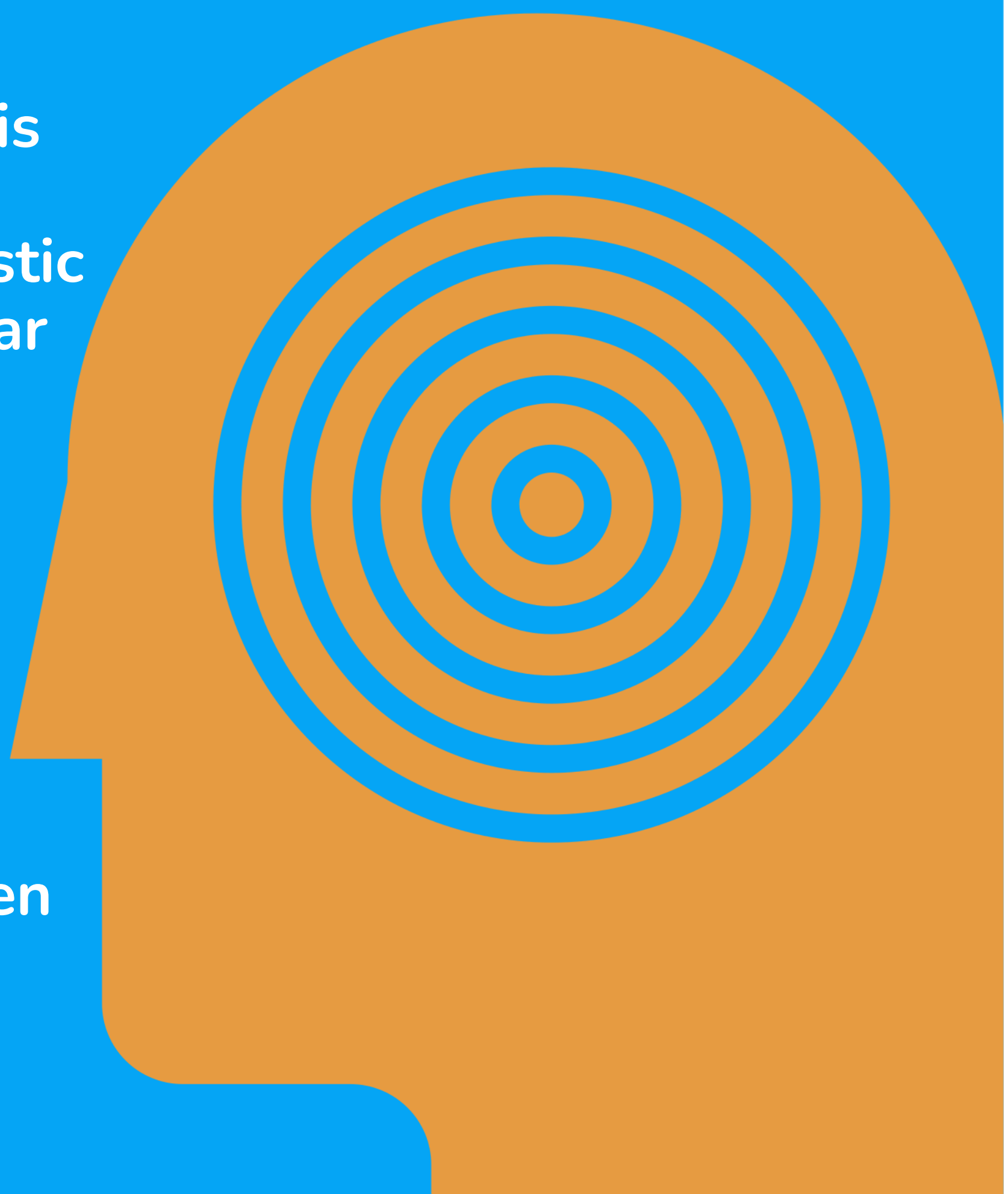

Scannez le code QR ou cliquez sur le lien et complétez le questionnaire.

https: //survey.kuleuven.cloud/814288?lang=fr

NEED sciensano KCE KU LEUVEN

Cette étude est le fruit d'une collaboration entre le KCE, Sciensano, et la KU Leuven. Elle s'inscrit dans le cadre du projet NEED

Plus d'info sur **https://health-needs.eu/index.php/fr/**

Cette étude a été approuvée par le Comité d'éthique de la Erasmus-ULB et UZ/KU Leuven

S70414

Trouble psychotique ?

Envie departiciper à une étude pour nous aider à mieux comprendre vos besoins non rencontrés? Votre voix compte !

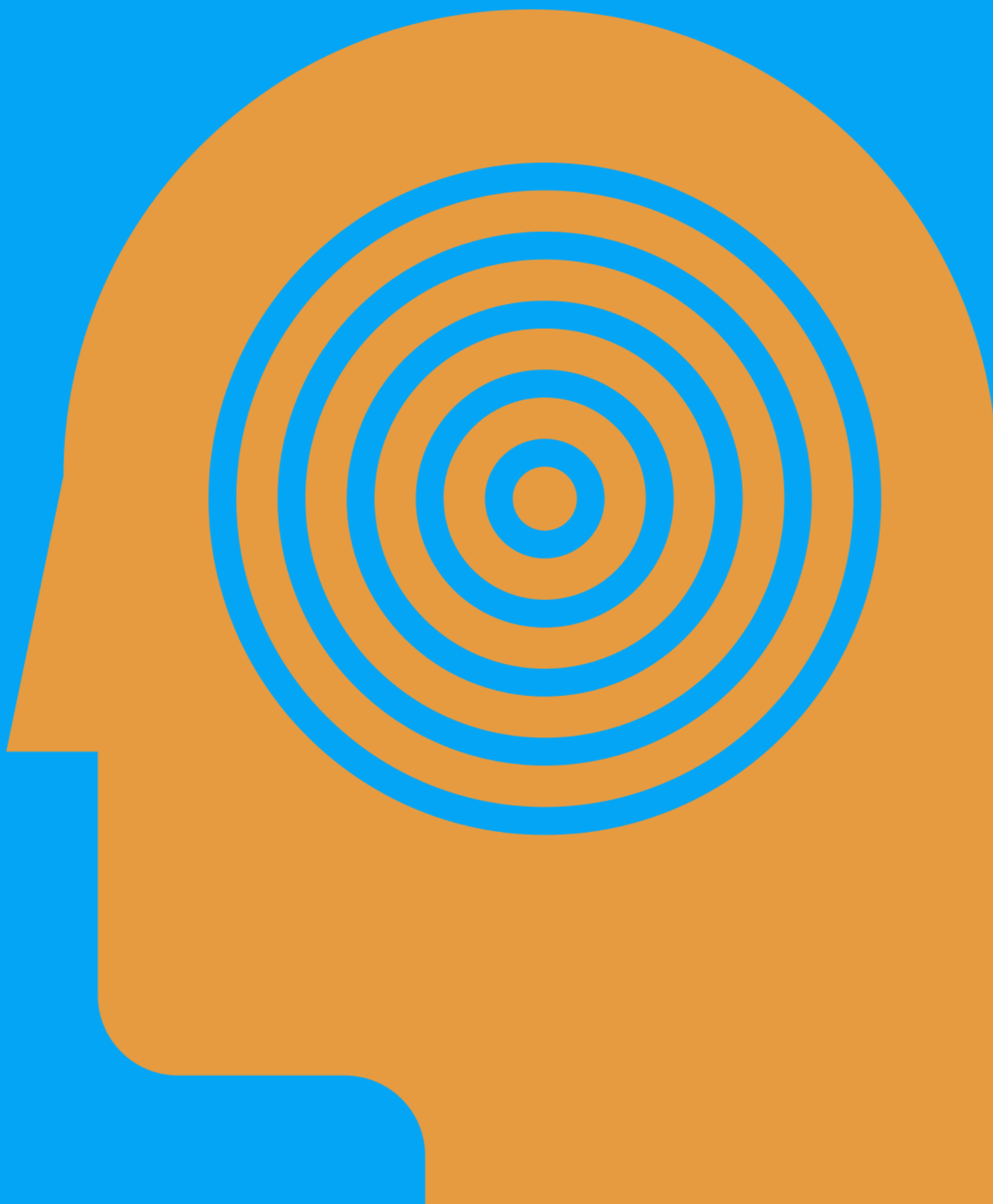

* Le questionnaire peut également être complété par un représentant légal si la personne n'est pas en mesure de le faire elle-même

Cette étude est le fruit d'une collaboration entre le KCE, Sciensano, et la KU Leuven. Elle s'inscrit dans le cadre du projet NEED

Cette étude a été approuvée par le Comité d'éthique de la Erasmus-ULB et UZ/KU Leuven

Plus d'info sur **https://health-needs.eu/index.php/fr/**

NEED sciensano KCE KU LEUVEN

S70414

Partagez votre éxperience et aidez-nous à améliorer les soins pour les patients atteints de troubles psychotiques

En participant à cette enquête, vous contribuerez à mettre en lumière les difficultés associées aux troubles psychotiques et à leur prise en charge, en vue d'améliorer les soins et les possibilités de traitement dans le futur

Cette étude est le fruit d'une collaboration entre le KCE, Sciensano, et la KU Leuven. Elle s'inscrit dans le cadre du projet NEED, qui vise à identifier les besoins de santé non rencontrés des patients et de la société, afin de mieux guider l'innovation dans les soins et les politiques de santé en fonction de ces besoins.

Compléter le questionnaire vous prendra 40 à 45 minutes. Vous avez jusqu'au 31/08/2025 pour participer.

Conditions de participation:

- Vous avez 18 ans ou plus
- Vous vivez en Belgique
- Vous comprenez le français ou le néerlandais
- Vous avez reçu un diagnostic de trouble psychotique (par ex. schizophrénie, trouble schizoaffectif, etc.)

Scannez le code QR et complétez le questionnaire

S70414

Psychotische problematiek?

Help ons om de onvervulde behoeften van mensen met een psychotische problematiek beter te begrijpen door deel te nemen aan onze enquête. Uw stem telt!

Deelname voorwaarden:

- U bent minstens 18 jaar
- U woont in België
- U bent Nederlands- of Franstalig
- U bent gediagnosticeerd met een psychotische problematiek (bv. schizofrenie, schizoaffectieve stoornis,...)

OF

- U bent de wettelijke vertegenwoordiger van iemand met een psychotische problematiek die niet in staat is om de enquête zelf in te vullen

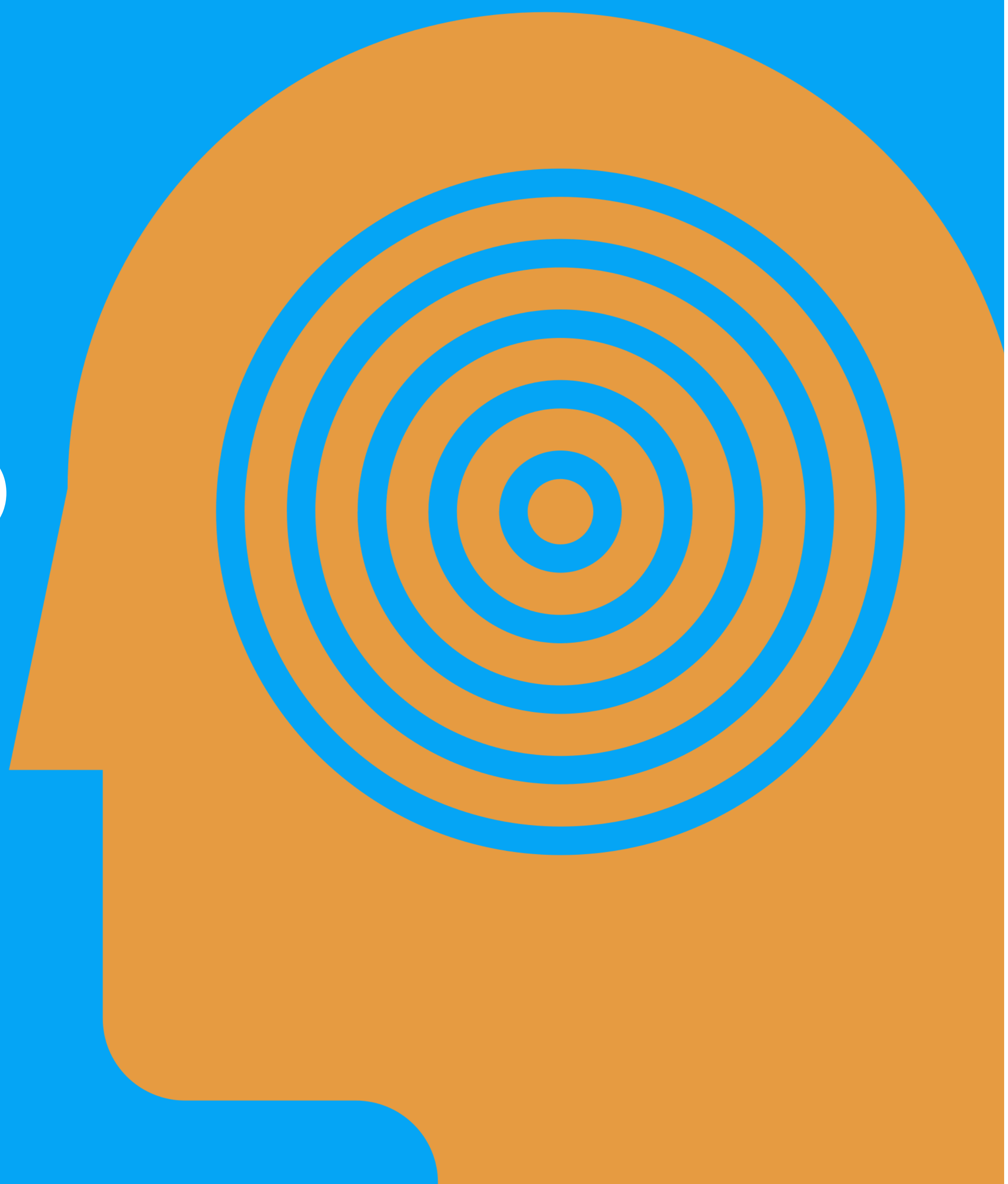

Scan de QR code of klik op de link en vul de vragenlijst in

https: //survey.kuleuven.cloud/814288?lang=nl

Deze studie is een samenwerking tussen het KCE, Sciensano, en KU Leuven. Ze maakt deel uit van het NEED-project

Meer informatie op **https://health-needs.eu/index.php/nl/**

Deze studie werd goedgekeurd door de Ethische commissie van Erasme-ULB en Onderzoek UZ/KU Leuven.

S70414

Psychotische problematiek?

Zin om deel te nemen aan een studie die ons helpt bij het begrijpen van uw onvervulde behoeften? Uw stem telt!

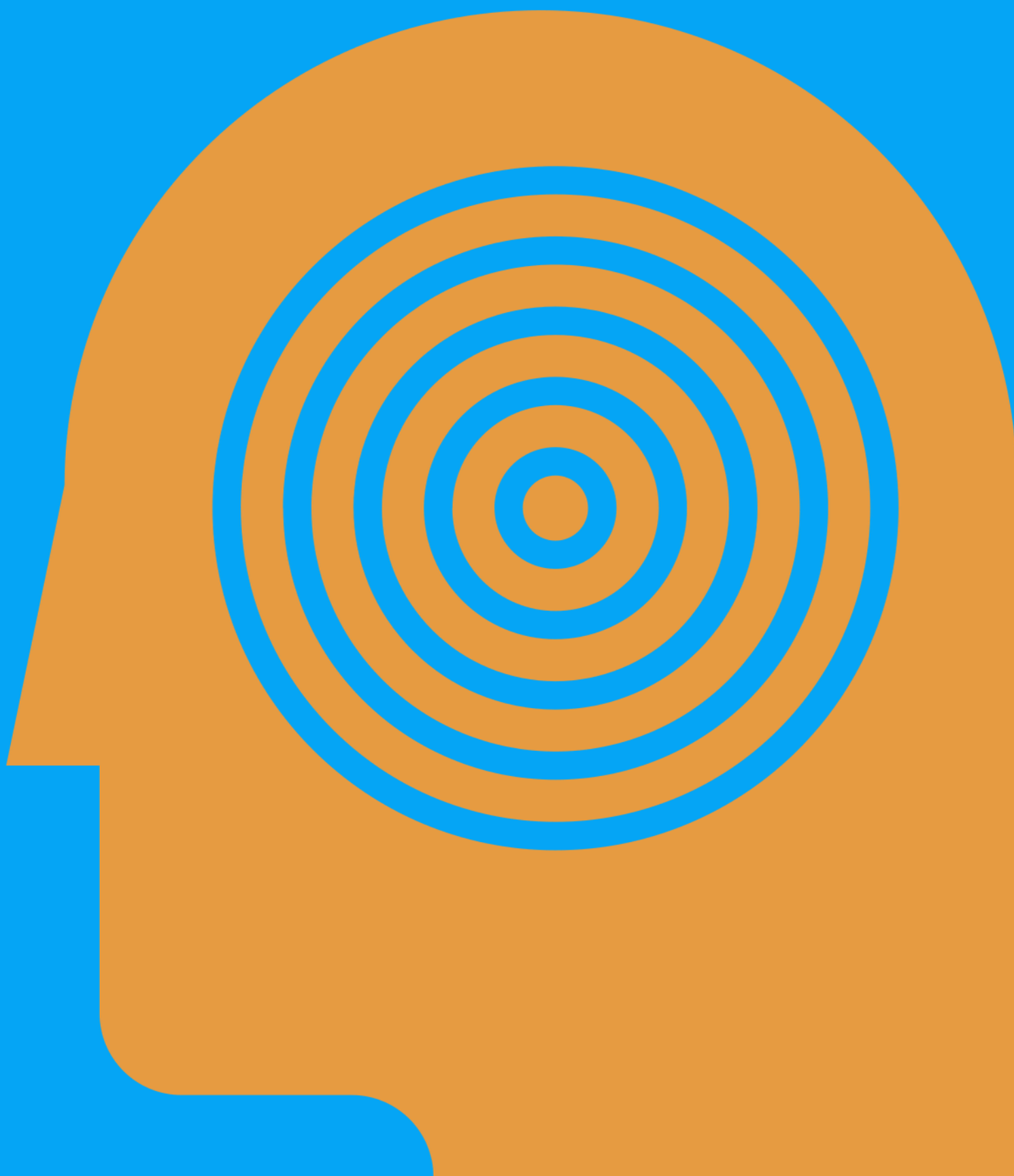

* De vragenlijst kan ook ingevuld worden door een wettelijke vertegenwoordiger indien de persoon niet in staat is om dat zelf te doen

Deze studie is een samenwerking tussen het KCE, Sciensano, en KU Leuven. Ze maakt deel uit van het NEED-project

Deze studie werd goedgekeurd door de Ethische commissie van Erasme-ULB en Onderzoek UZ/KU Leuven.

Meer informatie op **https://health-needs.eu/index.php/nl/**

NEED sciensano KCE KU LEUVEN

S70414

Deel jouw ervaring en help om de zorg voor patiënten met een psychotische problematiek te verbeteren

Door deel te nemen aan dit onderzoek zal u bijdragen aan het belichten van de moeilijkheden rond psychotische problematiek en hun behandeling, met als doel in de toekomst de zorg en behandelingsopties te verbeteren.

Deze studie is een samenwerking tussen het KCE, Sciensano en de KU Leuven. Ze maakt deel uit van het NEED-project, dat als doel heeft om de onvervulde gezondheidsbehoeften van patiënten en de samenleving in kaart te brengen, om innovatie in de zorg en het gezondheidsbeleid beter af te stemmen op deze behoeften.

Het invullen van de vragenlijst duurt 40 à 45 minuten. U heeft tot 31/08/2025 om deel te nemen.

Deelname voorwaarden:

- U bent minstens 18 jaar
- U woont in België
- U bent Nederlands- of Franstalig
- U bent gediagnosticeerd met een psychotische problematiek (bv. schizofrenie, schizoaffectieve stoornis,...)

Scan de QR-code om de vragenlijst in te vullen

S70414

Psychotic disorder?

Help us to better understand the unmet needs of individuals with psychotic disorders by participating in our survey. Your voice counts!

Conditions to participate:

- You are at least 18 years old.
- You live in Belgium.
- You are Dutch or French speaking.
- You have been diagnosed with a psychotic disorder (e.g. schizophrenia, schizoaffective disorder, ...).

OR

- You are the legal representative of someone with a psychotic disorder who is unable to complete the survey themselves.

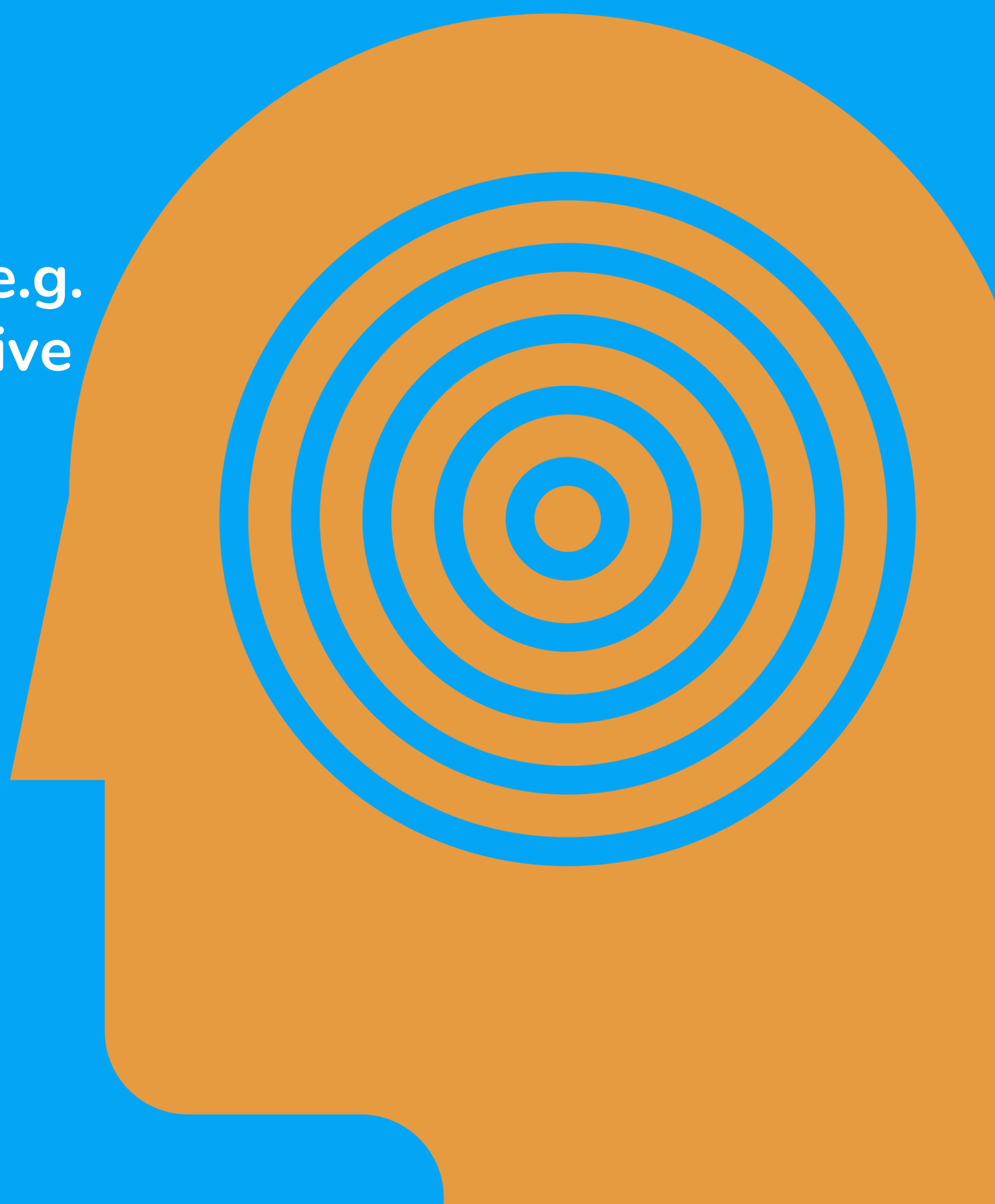

Scan the QR code or click on the link to complete the questionnaire.

https: //survey.kuleuven.cloud/814288?lang=nl

NEED sciensano KCE KU LEUVEN

This study is a collaboration between the KCE, Sciensano and KU Leuven and is part of the NEED project.

More information at **https://health-needs.eu/index.php/en/**

This study was approved by the Ethical Committee of Erasme-ULB and Research UZ/KU Leuven.

S70414

Psychotic disorder?

Interested in participating in a study that helps us understand your unmet needs? Your voice counts!

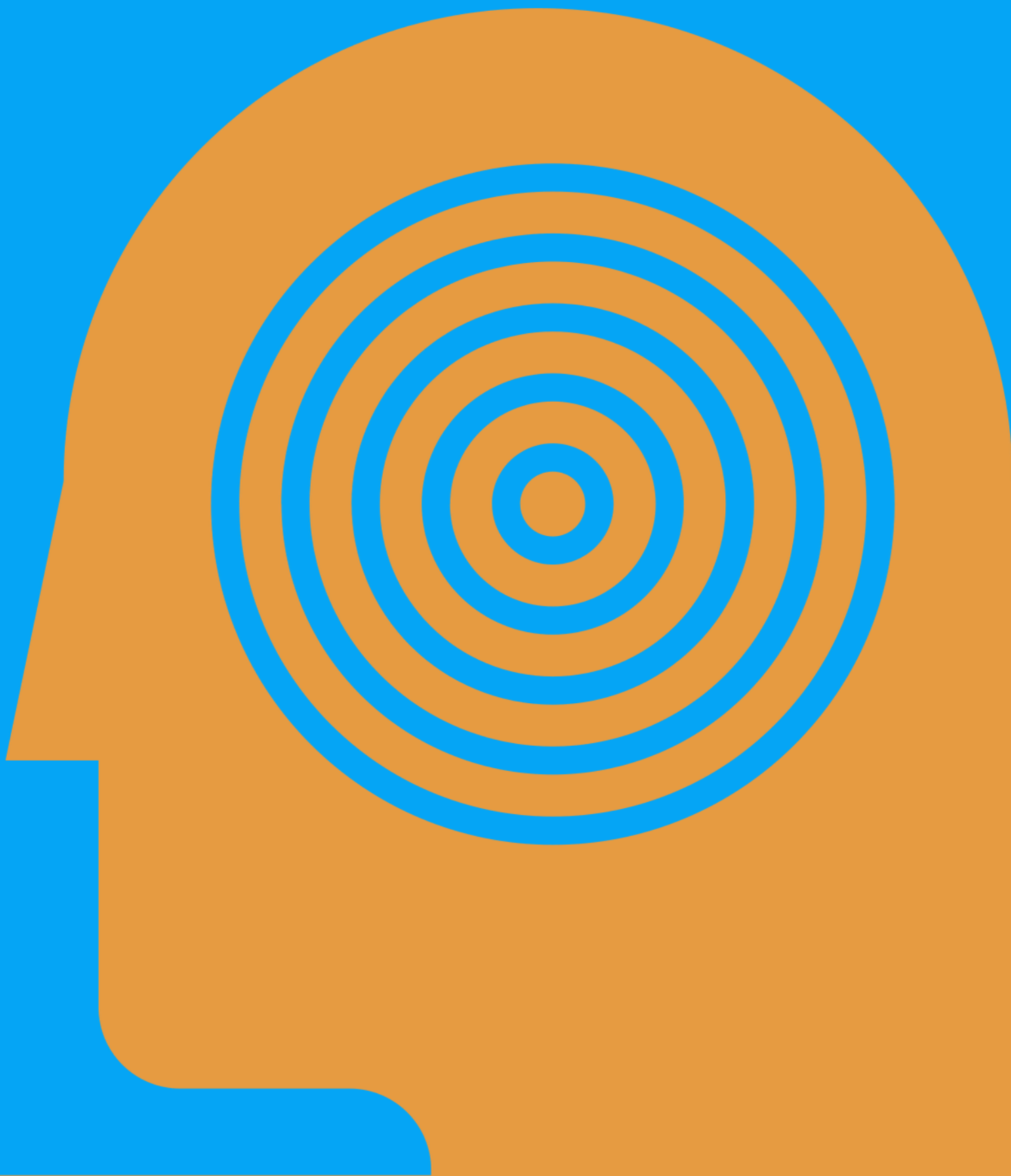

* The questionnaire cal also be completed by a legel representative if the person is unable to do so themselves.

This study is a collaboration between the KCE, Sciensano and KU Leuven and is part of the NEED project.

This study was approved by the Ethical Committee of Erasme-ULB and Research UZ/KU Leuven.

More information at **https://health-needs.eu/index.php/en/**

NEED sciensano KCE KU LEUVEN

S70414

Share your experience and help improve care for patients with a psychotic disorder.

By participating in this research, you will help shed light on the challenges associated with psychotic disorders and the treatment, with the aim of improving care and treatment options in the future.

This study is a collaboration between KCE, Sciensano and KU Leuven. It is part of the NEED project which aims to identify unmet health related needs of patients and society in order to better align innovation in healthcare and health policy.

Completing the questionnaire takes approximately 40 to 45 minutes. You can participate until 31/08/2025.

Conditions to participate:

- You are at least 18 years old.
- You live in Belgium.
- You are Dutch or French speaking.
- You have been diagnosed with a psychotic disorder (e.g. schizophrenia, schizoaffective disorder, ...).

Scan the QR-code to complete the questionnaire.
